# Structural basis of the strong cell‐cell junction formed by cadherin‐23

**DOI:** 10.1111/febs.15141

**Published:** 2019-12-11

**Authors:** Gayathri S. Singaraju, Amin Sagar, Anuj Kumar, Jesse S. Samuel, Jagadish P. Hazra, Malay K. Sannigrahi, Ragothaman M. Yennamalli, Ashish, Sabyasachi Rakshit

**Affiliations:** ^1^ Department of Chemical Sciences Indian Institute of Science Education and Research Mohali Punjab India; ^2^ Department of Physical Sciences Indian Institute of Science Education and Research Mohali Punjab India; ^3^ Department of Biotechnology and Bioinformatics Jaypee University of Information Technology Waknaghat India; ^4^ Institute of Microbial Technology (CSIR) Chandigarh India; ^5^ Centre for Protein Science Design and Engineering Indian Institute of Science Education and Research Mohali Punjab India

**Keywords:** atypical cadherins, cadherin‐23, cell–cell adhesion, single-molecule Forster resonance energy transfer, small‐angle X‐ray scattering

## Abstract

Cadherin‐23, a giant atypical cadherin, form homophilic interactions at the cell–cell junction of epithelial cells and heterophilic interactions with protocadherin‐15 at the tip links of neuroepithelial cells. While the molecular structure of the heterodimer is solved, the homodimer structure is yet to be resolved. The homodimers play an essential role in cell–cell adhesion as the downregulation of cadherin‐23 in cancers loosen the intercellular junction resulting in faster migration of cancer cells and a significant drop in patient survival. *In vitro* studies have measured a stronger aggregation propensity of cadherin‐23 compared to typical E‐cadherin. Here, we deciphered the unique trans‐homodimer structure of cadherin‐23 in solution and show that it consists of two electrostatic‐based interfaces extended up to two terminal domains. The interface is robust, with a low off‐rate of ~ 8 × 10^−4^ s^−1^ that supports its strong aggregation propensity. We identified a point mutation, E78K, that disrupts this binding. Interestingly, a mutation at the interface was reported in skin cancer. Overall, the structural basis of the strong cadherin‐23 adhesion may have far‐reaching applications in the fields of mechanobiology and cancer.

## Graphical abstract

Cadherin‐23, a long nonclassical cadherin, is known to mediate strong cell–cell junctions through homophilic interactions. Cadherin‐23‐mediated junctions are involved in cell–cell adhesion in a variety of epithelial tissues, and downregulation of this nonclassical cadherin in the context of cancer has been shown to promote faster migration of tumor cells. In this study, we used small‐angle X‐ray scattering (SAXS) in combination with in silico measurements to decipher the unique trans‐homodimer structure of cadherin‐23 that maintains the characteristically robust cell–cell junction.

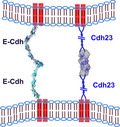

**Abbreviations**
AFM: atomic force microscopy
AUC: analytical ultracentrifugation
CD: circular dichroism
DLS: dynamic light scattering
MD: molecular dynamics
MTT: 3‐(4,5‐dimethylthiazol‐2‐yl)‐2,5‐diphenyltetrazolium bromide
RMSD: root mean square deviation
SAXS: small‐angle X‐ray scattering
SDS/PAGE: sodium dodecyl sulfate polyacrylamide gel electrophoresis
SE: sedimentation equilibrium
SEC: size exclusion chromatography
smFRET: single-molecule Forster resonance energy transfer
SMFS: single‐molecule force spectroscopy
SV: sedimentation velocity
TIRF: total internal reflection fluorescence

## Introduction

Cell–cell adhesion by classical cadherins, a subfamily of cadherin class of proteins, is well‐studied 1. Nonclassical cadherins that comprise more than 80% of cadherins also actively participate in cell–cell adhesion 2, 3. While the physiological significance of nonclassical cadherin‐mediated cell–cell junction is well recognized, little is explored on their molecular structures. Cadherin‐23 (Cdh23) is one of the giant nonclassical cadherins that forms strong intercellular junctions in nearly 90% of healthy epithelial tissues including the brain, lymph node, kidney, gastrointestinal tract, testis, and skin (The Human Proteome Atlas) 4, 5, 6. The strong junction that is mediated by the homophilic trans‐interactions of Cdh23 serves as metastasis suppressor for solid cancers including sarcoma, adrenocortical carcinoma, and cervical cancer (The Cancer Genome Atlas, TCGA) 6, 7. Interestingly, Cdh23 is also known for its strong binding with protocadherin‐15 (Pcdh15) at the tips of stereocilia in neuroepithelial cells where the complex serves as gating spring under sound stimuli 5, 6. While the structural detail of the heterophilic complex with Cdh23 is understood 8, the molecular details of the homophilic complex are not identified yet. Here, we aim to understand the molecular mechanism of the homophilic interactions of Cdh23 that mediate a robust cell–cell adhesion.

**Figure 1:**
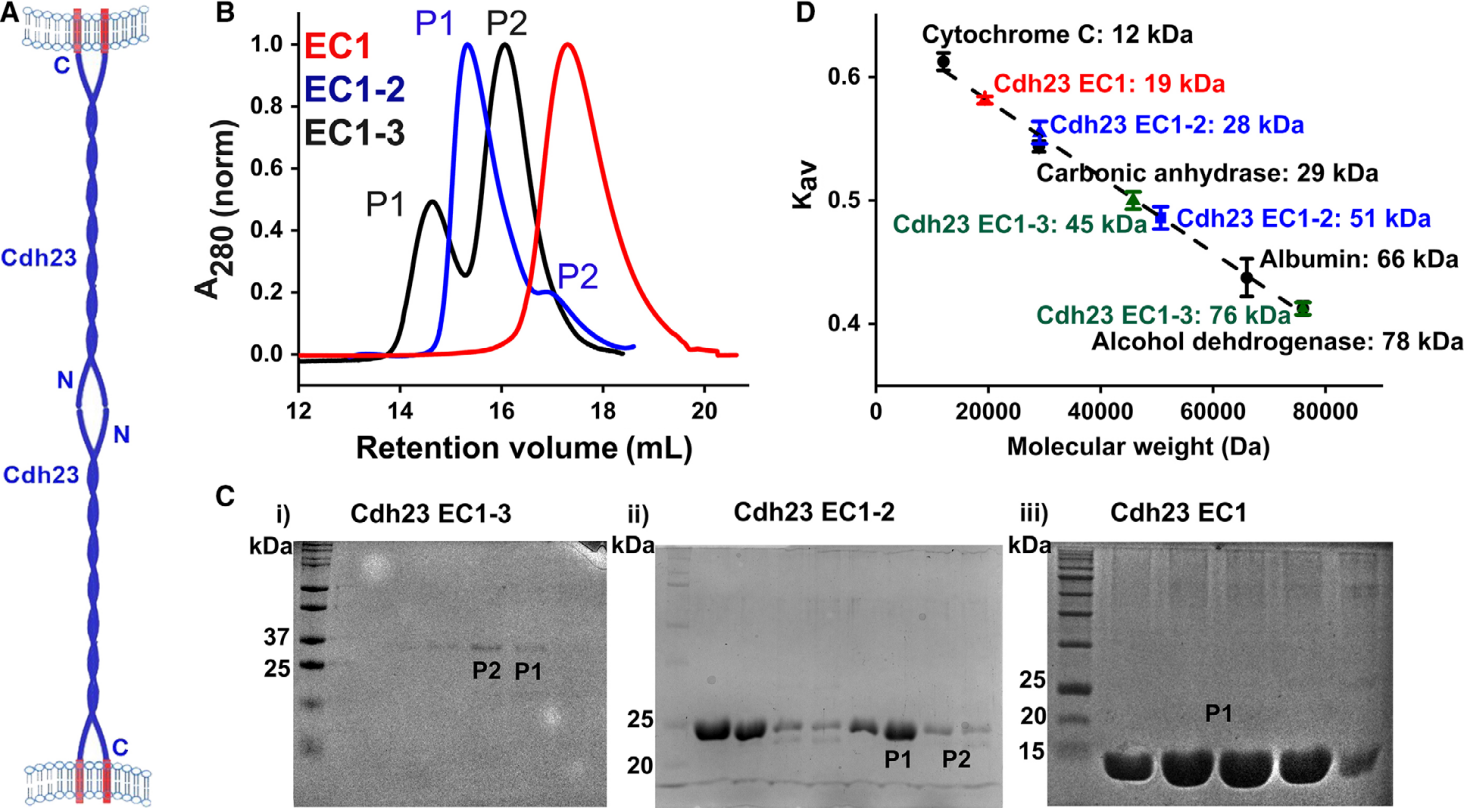
Identification and separation of Cdh23 homodimers in solution. (A) Schematic representation of Cdh23 proteins interacting from opposing cell surfaces to mediate the cell–cell junction. The schematic indicates that most of the EC domains of Cdh23 are engaged in cis‐interactions between proteins from the same cell surface, and only a few N‐terminal EC domains are available for trans‐interactions to mediate cell–cell junction. (B) Analytical SEC of Cdh23 EC1‐2 and Cdh23 EC1‐3 showed two peaks, whereas Cdh23 EC1 eluted in a single fraction. The first peak (P1) corresponded to a dimer, and the second peak (P2) corresponded to a monomer. (C) SDS/PAGE for all the SEC fractions of (i) Cdh23 EC1‐3, (ii) Cdh23 EC1‐2, and (iii) Cdh23 EC1 (A) along with molecular weight ladder is shown here. All the proteins appeared at their respective theoretical molecular weights: Cdh23 EC1‐3 (37 kDa) (i), Cdh23 EC1‐2 (26 kDa) (ii), and Cdh23 EC1 (15 kDa) (iii). (D) The linear plot between the partition coefficient and molecular weight of standard proteins maps the molecular weight of the proteins eluted at different fractions in the SEC (B). The apparent molecular weights for the two elutions, P1 and P2, of Cdh23 EC1‐2 (blue) and Cdh23 EC1‐3 (olive green), are marked in the calibration map. The single elution for Cdh23 EC1 is marked in red in the calibration curve. All the SECs were run in triplicate. The error bars represent the standard error of the mean (SEM) with *N* = 3.

Cdh23 comprises a cytosolic domain, a single‐pass transmembrane region followed by 27 extracellular (EC) domains, unlike just 5 EC domains in classical cadherins 9, 10, 11. However, similar to classical cadherins, Cdh23 causes cells to adhere using two types of homophilic interactions, *trans* and *cis* 4, 12. Electron tomography revealed a unique pattern for the cis‐homodimer of Cdh23: a pair of Cdh23 molecules aligned in the same orientation and intertwined to form a helical complex through interactions between all EC domains except few terminal domains 5. The domains at the N termini that are exposed outward are available for trans‐interactions (Fig. 1A). Using single‐molecule Forster resonance energy transfer (smFRET) and live‐cell aggregation assays, here we first identified the number of domains that participate in the trans‐dimerization. In relevance, the engagement of the two terminal domains in a heterophilic *trans*‐interaction with protocadherin‐15 (Pcdh15) was already reported in tip links in the inner ear 8.

Classical cadherins undergo homophilic *trans*‐interactions using the outermost terminal domain, EC1 13, 14. They first form an X‐dimer, a kinetically driven interaction and then converted to a thermodynamically stable strand‐swap dimer (S‐dimer) via an intermediate with the overlap of the linker region between two terminal domains 15, 16. Among nonclassical cadherins, desmosomal cadherins form an S‐dimer 17, whereas T‐cadherin and R‐cadherin form only X‐dimer 18. Cdh23, however, lacks the sequence determinants for either S‐ or X‐dimerization. Moreover, the EC1 domain of Cdh23 has several unique features: a 5 residue long 3_10_‐helix just prior to the A* β‐strand, an α‐helical loop connecting two β‐strands, and most strikingly, an additional Ca^2+^‐binding site toward the N terminus 19. Together, these features indicate a unique interface for Cdh23‐mediated homophilic *trans*‐interactions. It was therefore imperative to decipher the molecular details of the Cdh23‐mediated homophilic trans‐interactions.

Using small‐angle X‐ray scattering (SAXS) in combination with in silico measurements including docking and molecular dynamics, we identified the molecular structure of the trans‐homodimer of Cdh23. We verified the binding interface with a single point mutation that impaired the dimer. Finally, using analytical methods including ultracentrifugation (AUC) and single‐molecule force spectroscopy (SMFS) with an atomic force microscope (AFM), we estimated the thermodynamic and kinetic parameters of the homophilic complex.

## Results

### The homodimer of Cdh23 is a *trans*‐dimer interacting via the N termini of the two outermost domains (EC1‐2)

Two outermost domains of Cdh23 are known to form the trans‐heteromeric complex with Pcdh15 in tip links. In order to determine the number of EC domains required for the trans‐homodimerization, we expressed Cdh23 with varying lengths of EC domains: first domain alone (EC1), first two domains (EC1‐2), and first three domains (EC1‐3). All the constructs were expressed in *E. coli BL21 RIPL* following a reported protocol (Materials and methods) 19 and purified in two steps by Ni^2+^‐NTA‐based affinity followed by size exclusion chromatography (SEC) (Fig. 1B). We ran SEC for all the three constructs at a high concentration (100 µm) and observed two distributions in elutions, P1 and P2, for EC1‐2 and EC1‐3 and EC1 eluted as a monomer (Fig. 1B). We subsequently ran SDS/PAGE for all the eluted fractions and observed a single band at 26 kDa (Fig. 1C) for Cdh23 EC1‐2 and 38 kDa for Cdh23 EC1‐3, suggesting that the P2 and P1 corresponded to the monomer and a higher‐order association, respectively. To determine the molecular weights of the elutions, we developed a calibration curve for SEC with standard proteins of varying molecular weights under the same conditions (Fig. 1D). From the standard curve, we then estimated the apparent molecular weights of proteins eluted at P2 and P1 fractions. The apparent molecular weights corresponded to 27 and 51 kDa for Cdh23 EC1‐2 and 45 and 76 kDa for Cdh23 EC1‐3, respectively. These estimated molecular weights corroborated with the theoretical monomer and dimer molecular weights, 26 and 52 kDa for Cdh23 EC1‐2 and 39 and 79 kDa for Cdh23 EC1‐3, respectively, confirming a dimer in the higher‐order association. The negligible variations in the apparent molecular weights, as opposed to the theoretical values, may arise from the differences in shapes of Cdh23 than the standard globular proteins. Further, we noticed significantly higher intensity for P1 than P2 for Cdh23 EC1‐2 and reversed for Cdh23 EC1‐3, though the loading concentration of proteins was the same for both the SEC runs (Fig. 1B). Thus, the higher intensity of P1 than P2 for Cdh23 EC1‐2 is indicative of the highest binding affinity for Cdh23 EC1‐2 toward homodimerization than the other constructs.

Next, we performed smFRET to decipher the orientations and the extent of overlap of the constituent monomers in the dimer. We used Cdh23 EC1‐2 and Cdh23 EC1‐3 for the experiment and not Cdh23 EC1, as Cdh23 EC1 showed the weakest affinity toward dimerization in the SEC. Since we performed smFRET on a glass coverslip using a total internal reflection fluorescence microscopy (TIRFM), we covalently anchored the C terminus of the proteins, Cdh23 EC1‐2, and Cdh23 EC1‐3 individually, to the coverslips using sortagging chemistry as reported 20, 21. All protein constructs were recombinantly modified with the sortase recognition sequence (–LPETGSS) at the C terminus. Prior to the surface attachment, proteins were modified with a donor (D) dye, Cyanine3 (Cy3, λ_ex_ = 545 nm; λ_em_ = 560 nm), at the N terminus. For N‐terminal modification, we recombinantly mutated the valine at position 3 to cysteine (V3C) and attached Cy3 using the thiol–maleimide Michael addition reaction. Thus, irrespective of the Cdh23 constructs, the surface‐anchored proteins always had donor dyes at the N terminus. Next, the protein modified surface was incubated with the second batch of proteins for 30 min for the homodimers to form, followed by vigorous washing to remove nonspecific attachments. The protein in solution was tagged with acceptor (A) dye, Cyanine5 (Cy5, λ_ex_ = 645 nm; λ_em_ = 660 nm), either at the C terminus or N terminus. The N‐terminal modification was done using V3C protein constructs, whereas the C‐terminal modification was done using sortagging followed by the thiol–maleimide Michael addition (Materials and methods). For all the constructs, the final dye to protein ratio was maintained at 1 : 1.

**Figure 2:**
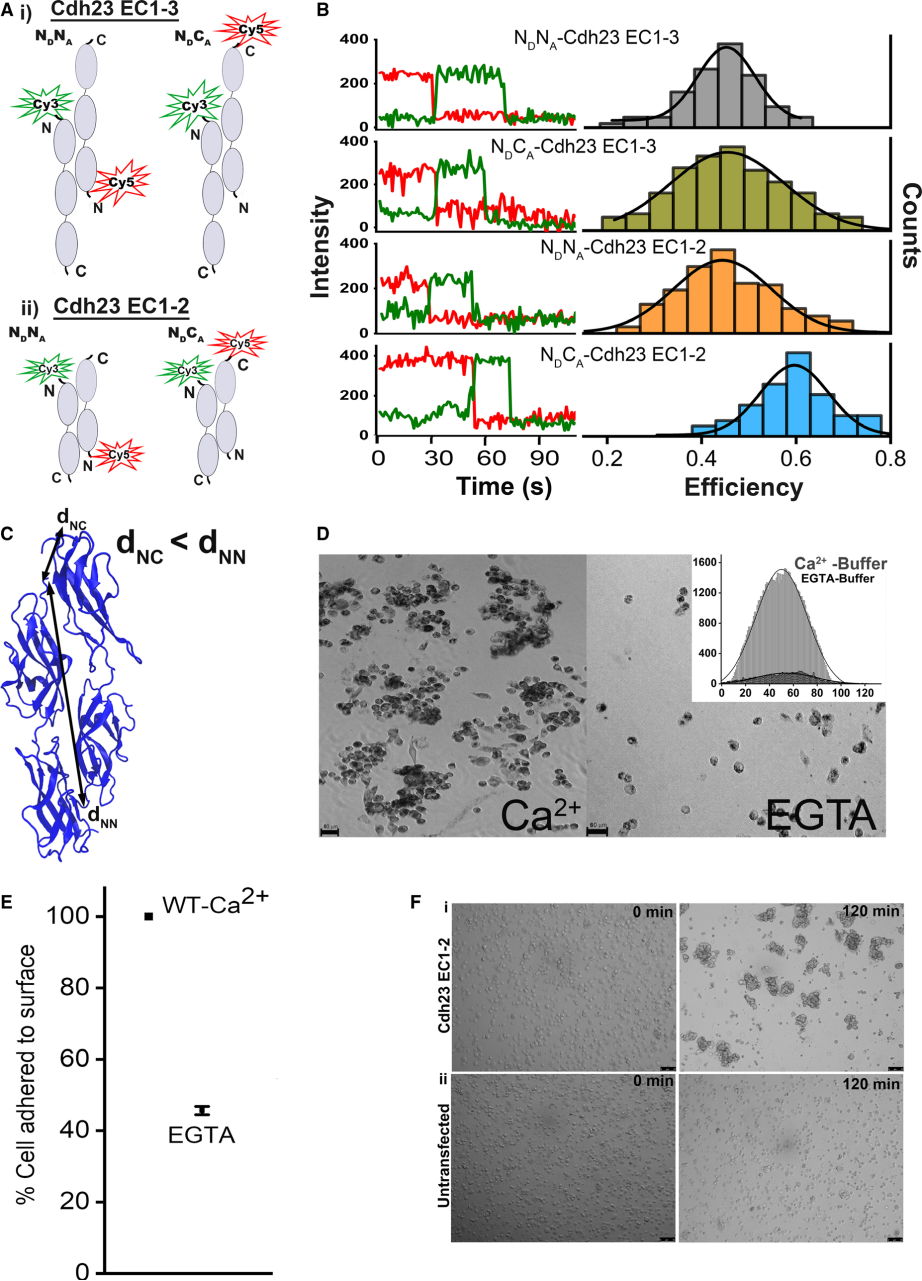
Homodimerization of cadherin‐23 in the *trans*‐conformation. (A) The probable orientations of the proteins, (i) Cdh23 EC1‐3 and (ii) Cdh23 EC1‐2, in the dimeric forms are represented schematically. The conformations are named as N_D_N_A_ or N_D_C_A_ according to their dye‐labeled terminals. (B) Left panel represents the time traces of the fluorescence intensities for the donor (D, green) and acceptor (A, red) dyes for four different complexes, two with Cdh23 EC1‐2 and the rest two with Cdh23 EC1‐3. For both sets of homodimers, the complexes are marked differently as N_D_N_A_ and N_D_C_A_ as per the labeling terminals with the donor (D) and acceptor (A) dyes. For each set, we analyzed 25 spots that showed FRET (N = 25). All the FRET experiments were repeated twice. The corresponding FRET efficiency distributions are plotted in the right‐side panel. (C) Schematic depiction of the distances between the terminals of the interacting proteins, d_NC_ and d_NN_. (D) Left, Pull‐down of live A549 cells expressing Cdh23 (Variant‐1) endogenously, by the surface‐anchored Cdh23 EC1‐2 proteins in the presence of Ca^2+^ buffer. Right, The cells detached upon chelating out Ca^2+^ ions using EGTA (scale bar: 60 µm). The inset shows the histogram of the number of cells adhered to the surfaces in the presence (gray) and absence (black) of Ca^2+^. The number of cells was estimated from the grayscale intensity of the images using ImageJ software. (E) The scattered plot represents the percentage of adhered A549 cells to the surfaces, estimated from the MTT assay. The percentages are measured from five different surfaces for each sample, and each experiment was repeated 3 times, which makes *N* = 15. The error bars represent the SEM with *N* = 15. (F) (i) Time‐dependent cell aggregation of HEK‐293 cells transiently transfected with Cdh23 EC1‐2 is captured using bright‐field imaging along with the control of (ii) untransfected HEK‐293 cells (scale bar: 75 µm). For both experiments, the cells were transfected with Cdh23 siRNA to stop the expression of endogenous Cdh23. The images were captured over five different surfaces, and the experiments were repeated 3 times, which makes *N* = 15.

smFRET was measured for two different combinations of each protein construct, N_D_N_A_ and N_D_C_A_. N_D_N_A_ is N‐terminal donor (D) with N‐terminal acceptor (A) dye, and N_D_C_A_ is N‐terminal donor (D) with C‐terminal acceptor (A) dye. Altogether, we have four such combinations, N_D_N_A_ and N_D_C_A_ for Cdh23 EC1‐3 homodimers (Fig. 2A(i)) and Cdh23 EC1‐2 (Fig. 2A(ii)) homodimers. We excited the donor molecules with a 532 nm laser and subsequentially monitored emissions in donor and acceptor channels for 400 s using an EMCCD (Materials and methods). We used isms software (The Birkedal lab, Aarhus Universitet) for the analysis of FRET traces at each colocalized spot of single donor and single acceptor (Materials and methods) 22. The FRET efficiency (*E*_FRET_) was estimated from the intensity ratios between acceptor and donor and plotted as distributions (Fig. 2B, left panel). The unimodal distribution of *E*_FRET_ for all the combinations indicates a single conformational population of the dimer (Fig. 2B, right panel). We used the peak maxima of the distributions as most probable efficiency ($E_{\text{FRET}}^{\text{mp}}$) and estimated the donor–acceptor separations for each $E_{\text{FRET}}^{\text{mp}}$.

The maximum *E*_FRET_ was measured for the N_D_C_A_ combination of Cdh23 EC1‐2 homodimers with an $E_{\text{FRET}}^{\text{mp}}$ of 0.60 ± 0.007, indicating the closest proximity between the N terminus and C terminus of Cdh23 EC1‐2 constructs (Fig. 2C). Interestingly for all other combinations, we measured lower $E_{\text{FRET}}^{\text{mp}}$ values with negligible differences. The closer association of the N_D_C_A_ than the N_D_N_A_ termini in Cdh23 EC1‐2 confirms the trans‐conformation of the dimer (Fig. 2C). The lower $E_{\text{FRET}}^{\text{mp}}$ for the N_D_C_A_ combination of Cdh23 EC1‐3 than Cdh23 EC1‐2 indicates that the overlap extends only up to EC1‐2 domains. Finally, no significant differences in the *E*_FRET_ distributions of Cdh23 EC1‐3 for N_D_C_A_ and N_D_N_A_ combinations support an extended overlap of the EC1‐2 domains in the trans‐homodimer as depicted in the models (Fig. 2A, Table S1). The comparable $E_{\text{FRET}}^{\text{mp}}$ of N_D_N_A_ for Cdh23 EC1‐2 with Cdh23 EC1‐3 further supports the model of extended overlap between EC1‐2 domains in the trans‐homodimer (Fig. 2A(i) and Table S1). Thus, we conclude from our smFRET measurements and SEC results that Cdh23 forms *trans*‐homodimer with the two outermost domains (EC1‐2) alone. In all our following experiments, we used Cdh23 EC1‐2 only, unless mentioned.

### Cdh23 EC1‐2 can mediate cell–cell adhesion

A549 cells endogenously express Cdh23 (full‐length, variant‐1) 6. To check the functional significance of Cdh23 EC1‐2 concerning the full‐length construct, we measured the *in vitro* binding of A549 live cells on Cdh23 EC1‐2‐modified surfaces. For the cell adhesion experiment, C terminus of the Cdh23 EC1‐2 proteins was covalently attached on the coverslip using sortagging and then incubated with ~ 10^4^ numbers of A549 live cells for 2 h, followed by repeated gentle washes with a buffer containing 2 mm of Ca^2+^‐ions. We subsequently imaged the coverslips under bright field and observed a large number of cells remained adherent to the surface (Fig. 2D, left panel). Since the cadherin‐mediated interactions are Ca^2+^‐dependent, as a control for the specificity of the cell surface interactions, we monitored the number of cells adhered to surfaces after washing with Ca^2+^‐free EGTA buffer (Fig. 2D, right panel). We noticed ~ 86% reduction in the number of cells adhered to the surface (Fig. 2D, Inset) as expected. We further quantified the number of live cells adhered to the surface by colorimetric MTT assay and measured 55.8% fewer cells for EGTA‐washed coverslips (Fig. 2E). These studies corroborate with the smFRET results, supporting that EC1‐2 domains of Cdh23 are enough to mediate the Ca^2+^‐dependent cell–cell adhesion. We extended the demonstration from the aggregation of live cells (HEK‐293) transfected transiently with Cdh23 EC1‐2 23. Untransfected HEK‐293 cells were used as a control. For both, cells were pretreated with Cdh23‐specific siRNA to silence the endogenous expression of Cdh23. We observed that cells transfected with Cdh23 EC1‐2 formed aggregates within 120 min (Fig. 2F(i)), while the untransfected HEK‐293 cells did not show any aggregates (Fig. 2F(ii)).

### SAXS envelope portrays a compact extended‐handshake conformation for the dimer

**Figure 3:**
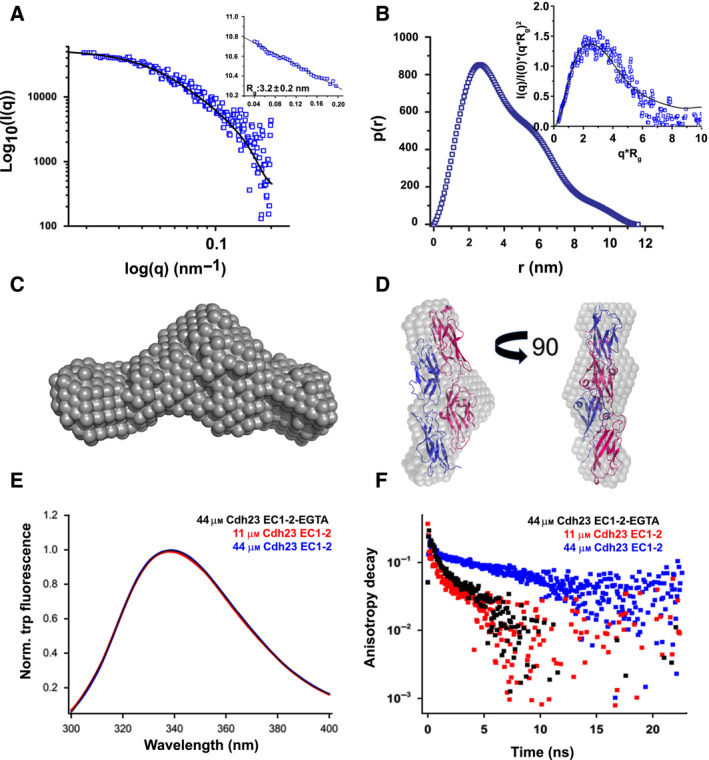
Cadherin‐23 dimer forms an extended‐handshake conformation. (A) SAXS profile of the Cdh23 EC1‐2 dimer is plotted as the scattering intensity I(q) vs. the scattering vector (q) on a log–log scale. The solid black line represents the theoretical SAXS profile computed from the proposed model of the dimer. The average scattering intensity is collected by exposing the sample to radiation for 20 min in 3 cycles. The acquisition was performed for 5 different batches of proteins, which makes *N* = 15. The linear nature of the Guinier plot (ln(*I*(*q*)) vs. q^2^) for the low q region is marked (inset, bottom, left). (B) The *p*(*r*) distribution for the dimer (blue) showing the maximum linear dimensions (*D*_max_) to be 11.6 nm. The normalized Kratky plot (*I*(*q*)/*I*(0)*(q*R_g_)^2^ of the experimental SAXS profile superimposed with that of the theoretical SAXS profile of the proposed dimer is shown in the inset. (C) The average *ab initio* model obtained from DAMMIN is shown for the dimer. (D) The statistically verified and rank‐1 PatchDock structure (PDB: 2WHV) of the dimer (blue) was fitted to the SAXS envelope (gray) for the dimer and shown with two orthogonal perspectives. (E) Steady‐state fluorescence was measured for Cdh23 EC1‐2 (λ_ex_ = 295 nm; λ_em_ = 338 nm) to identify the change in the W66 environment in different protein states: monomeric (blue and black) and dimeric (red). The reproducibility of the result was checked by repeating the experiments 3 times with 3 different batches of protein. (F) Time‐resolved fluorescence anisotropy decays of *W66* in Cdh23 EC1‐2 were recorded at two concentrations of protein, 11 µm (red) to 44 µm (blue), in Ca^2+^‐buffer. Increasing protein concentrations increase the dimer population in the solution. The black dots represent the fluorescence anisotropy decays of 44 µm Cdh23 EC1‐2 in 1 mm EGTA where the protein is expected to be in monomeric form exclusively. We confirmed the reproducibility of the result by repeating all the experiments 3 times for each concentration, both 11 and 44 µm, using different batches of protein.

To reveal the shape of the Cdh23 EC1‐2 *trans*‐homodimer in solution, we performed SAXS measurements with proteins at 10 mg·mL^−1^ (Fig. 3A), for a q‐range of 0.01 to 0.45 Å^−1^ using SAXSpace instrument (Anton Paar GmbH, Graz, Austria) (Table S2). The monodispersity of the sample was verified from the linearity of the Guinier plot (Fig. 3A, inset). The folding of the proteins was confirmed from the strong agreement between the normalized Kratky plots (*I*(*q*)*(q*R_g_)^2^/*I*(0) vs q*R_g_) of the experimental data and the theoretical SAXS profiles of the crystal structures/models (Fig. 3B, inset). It is pertinent to mention here that the peaks of the normalized Kratky plots deviate from the value of 1.73. This can be attributed to the rod‐like shape of the protein, as the theoretical SAXS profile of the docked model also showed the same profile (Fig. 3B, inset) 24. The molecular weight was estimated as 54 kDa from the volume of correlation (*V*_c_) 25 and 60 kDa using lysozyme as standard. From the indirect Fourier transformation of the SAXS profile using GNOM 26, we generated the pair distribution function (*P*(*r*)), which provided the frequency of the interatomic vectors inside the predominant shape of the proteins in real space(*r*). *P*(*r*) curves estimated for dimer showed a maximum linear dimension (*D*_max_) and *R*_g_ values of 11.6 nm and 3.2 ± 0.2 nm, respectively (Fig. 3B). The *D*_max_ further supports the extended‐handshake conformation for the dimer as predicted from smFRET. The peak position and shoulder profile of the computed *P*(*r*) supported the multidomain shape of Cdh23 connected by a nonflexible linker.

To obtain a three‐dimensional shape of the dimer, we generated ten independent dummy residue models using DAMMIF 27, averaged using DAMAVER, refined using DAMMIN 28, and compared with each other by calculating the normalized spatial discrepancy (NSD). NSD is a measure of the similarity in the shapes of the models with a value of < 1 indicating that the models are relatively similar and values above 1 indicating that the models are systematically different from each other. The mean NSD between the 10 models was 0.602 with a standard deviation of 0.027, indicating that all the models were very similar to each other. We averaged all ten models and obtained an envelope with dimensions of 14(L) × 5.76(W) × 4.1(H) nm (Fig. 3C). We repeated the modeling protocol several times and validated the protocol as robust and reproducible.

### Homodimerization of Cdh23 EC1‐2 is not mediated by tryptophan

Next was to identify a dimer structure that could fit the SAXS envelope. We first considered the W‐conformation of Cdh23 dimer proposed previously 19. It was predicted that Cdh23 EC1‐2 might form a *trans*‐homodimer through π‐stacking of the indole ring of the sole tryptophan at 66th position (W66). The driving force behind such W‐mediated interactions is the switch of the W‐environment from hydrophilic to more hydrophobic. Since W is known to feature a solvatochromic shift in fluorescence, we designed experiments to probe the W‐emission of the monomer and dimer and decipher its role in dimerization. Accordingly, we monitored the steady‐state emission of W66 (λ_ex_ = 295 nm). As the protein concentration increased from a monomer concentration to beyond the *K*_D_ (~18 µm) of the dimer (Fig. 3E), we did not observe any shift in W‐emission, excluding its possible involvement in dimerization. Further, we examined the mobility of W66 in the monomer and dimer using time‐resolved fluorescence anisotropy. Trp in a protein can have two rotational components, a fast rotation around its axis and a relatively slower rotation along with the protein. Dimerization via the π‐stacking of the W residues is expected to constrain the free local rotation around its axis and therefore delay the anisotropy decay of the fast component. We probed this anisotropy decay of W66 at two concentrations of proteins ranging from monomer populated solution to dimer dominated solution (Fig. 3F) and observed no change in the decay rate of the faster components This indicated a lack of constraints on the local mobility of W66 and thus confirming that W66 did not play a role in dimerization. However, we observed a significant increase in the decay for the global rotation of W66 upon dilution indicating a decrease in overall size. The decay at low concentration matches with the global rotation of Cdh23 in the presence of EGTA, which is expected to be in the monomeric form (Fig. 3F and Table S3).

Once we confirmed the W‐conformation cannot be the dimer, we focused on searching for new structures by docking Cdh23 EC1‐2 (PDB ID: 2WHV) using PatchDock. Of the first ten models based on dock‐scoring, seven models showed docking through the N terminus of the protein and the others through the C terminus. We considered these seven models and superimposed them with the SAXS envelope using SUPCOMB, which showed comparable NSD values varying from 0.7 to 0.8 for all the structures. However, rank 1 appeared as the best fit model from the *z*‐tests based on *R*_g_ and FRET‐based end‐to‐end distances (*P* = 0.20) (Fig. 3D and Table S4).

**Figure 4:**
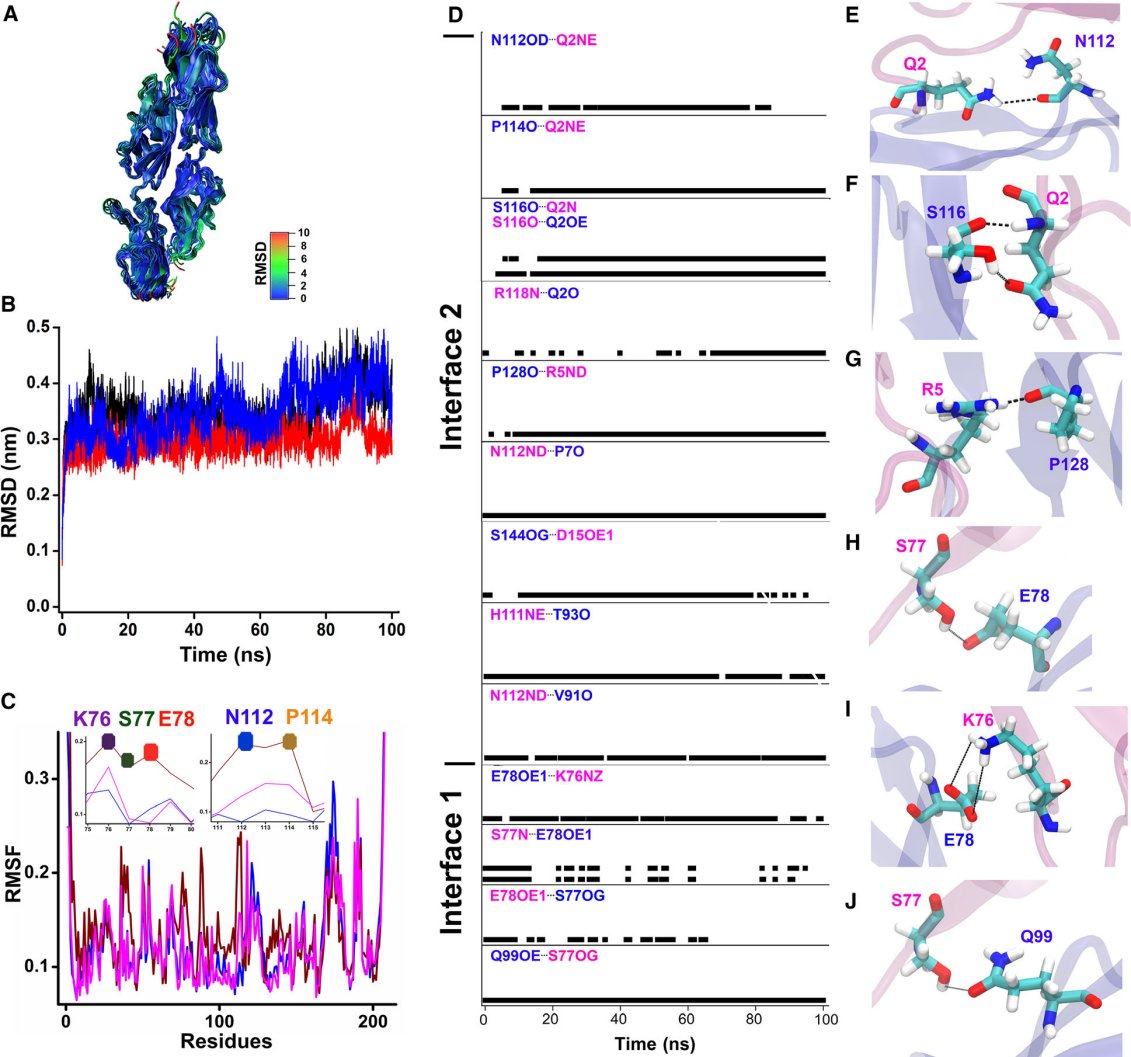
Survey of the H bonds that are crucial for dimerization. (A) Superimposed snapshots of the Cdh23 EC1‐2 dimer at 10‐ns intervals of the entire trajectory of the MD run are shown as a visual guide to the RMSD plot. (B) RMSD for the rank‐1‐docked Cdh23 EC1‐2 dimer is shown for 3 independent MD runs of 100 ns. (C) The RMSF is shown to compare the average fluctuations (*N* = 3) of all residues in chain A (magenta) and chain B (blue) of the dimer with the monomer (wine red). The zoomed plots in the inset highlight the RMSF of the interacting residues (left: K76 (purple), S77 (green), E78 (red) from interface 1 and right: N112 (blue) and P114 (yellow) from interface. (D) The plot shows the survival probabilities of the H bonds over time between different residues. From bottom to top, the interaction residues are divided into interface 1 and interface 2. The residues in pink are from chain A, and the blue residues are from chain B of the dimer. (E–J) The images show the relative position of the residues involved in H bonding in enlarged diagrams between two chains, Chain A (magenta) and Chain B (blue): (E) H bonds between N112‐Q2; (F) Q2–S116; (G) R5‐P128; (H) E78‐S77; (I) K76‐E78; and (J) Q99‐S77. The black dots represent the H bonds.

**Figure 5:**
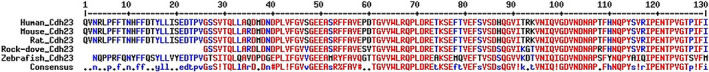
Sequence alignment indicates that the residues forming interface one are conserved in human, zebrafish, rat, and mouse. Sequence alignment of Cdh23 protein is compared among human (UniProt accession no. Q9H251), mouse (UniProt accession no. Q99PF4), rat (UniProt accession no. P58365), rock dove (UniProt accession no. R7VWU6), and zebrafish (UniProt accession no. Q6QQE1) using an online tool, Kalign2 1.

**Figure 6:**
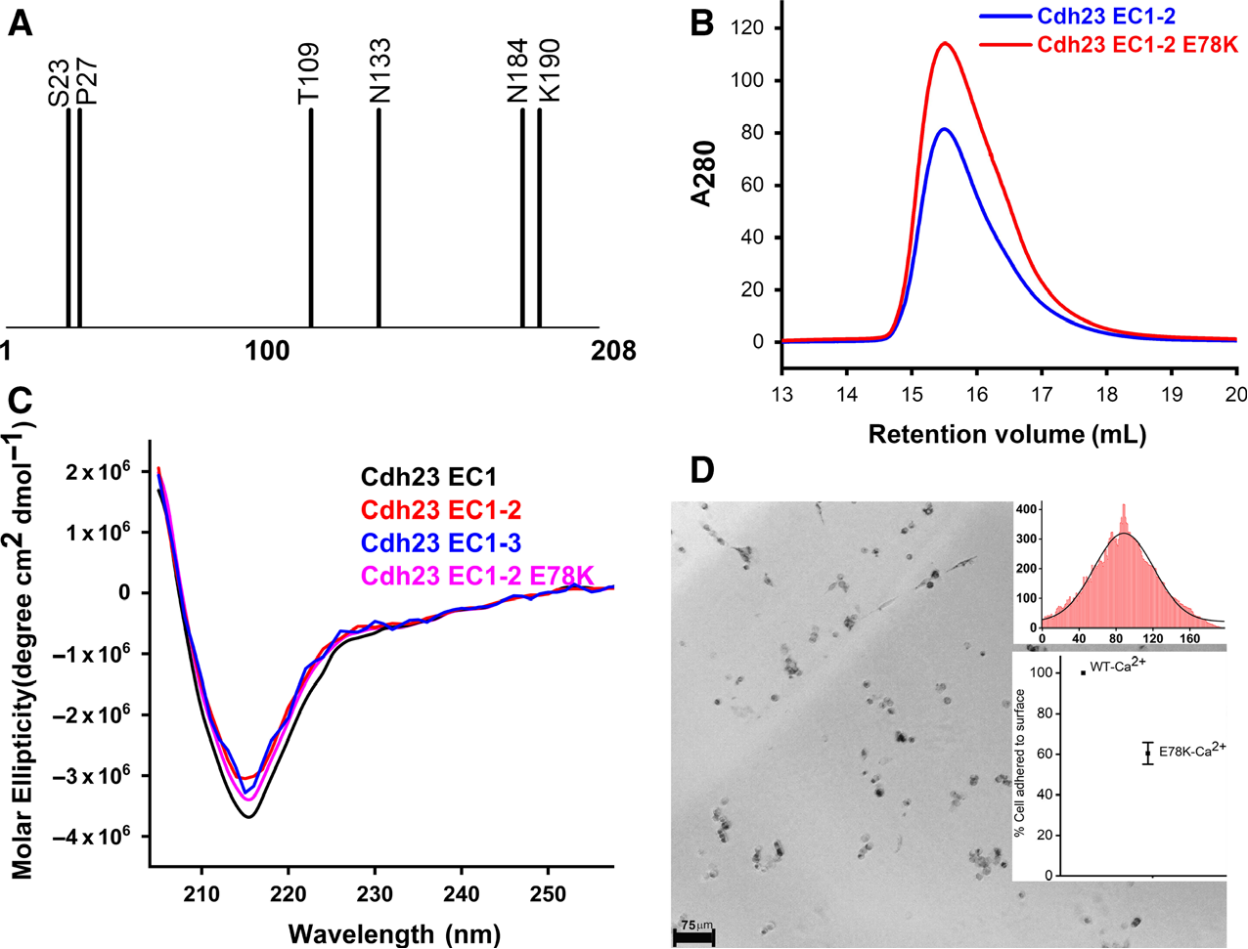
E78K of Cdh23 may serve as a contact‐inhibition mutant. (A) The plot reveals the putative N‐glycosylation sites for Cdh23 EC1‐2 at N133, N184, K190, and O‐glycosylation at S23, P27, T109 predicted using http://www.cbs.dtu.dk/services/NetNGlyc/ and http://www.cbs.dtu.dk/services/NetOGlyc/. (B) Comparative SEC plots of 50 µm Cdh23 EC1‐2 WT (blue) and 100 µm Cdh23 EC1‐2 E78K (red) show a single elution for both the proteins. (C) Molar ellipticity of all Cdh23 EC constructs (WT and mutants) is overlaid to highlight their structural uniformity. (D) The live‐cell images of the number of A549 cells expressing Cdh23 (Variant‐1) adhered to a surface coated with Cdh23 EC‐2 (E78K) in the presence of Ca^2+^ were captured (scale bar: 75 µm). The top inset shows the histogram of the number of cells estimated from the grayscale intensity of the images using ImageJ software (red). Bottom inset shows the percentage of adhered A549 cells to the surfaces modified with Cdh23 EC1‐2 WT and Cdh23 EC1‐2 E78K, estimated from the MTT assay. The percentages were measured from five different surfaces for each sample, and each experiment was repeated 3 times, which makes *N* = 15. The error bars represent the SEM with *N* = 15.

**Figure 7:**
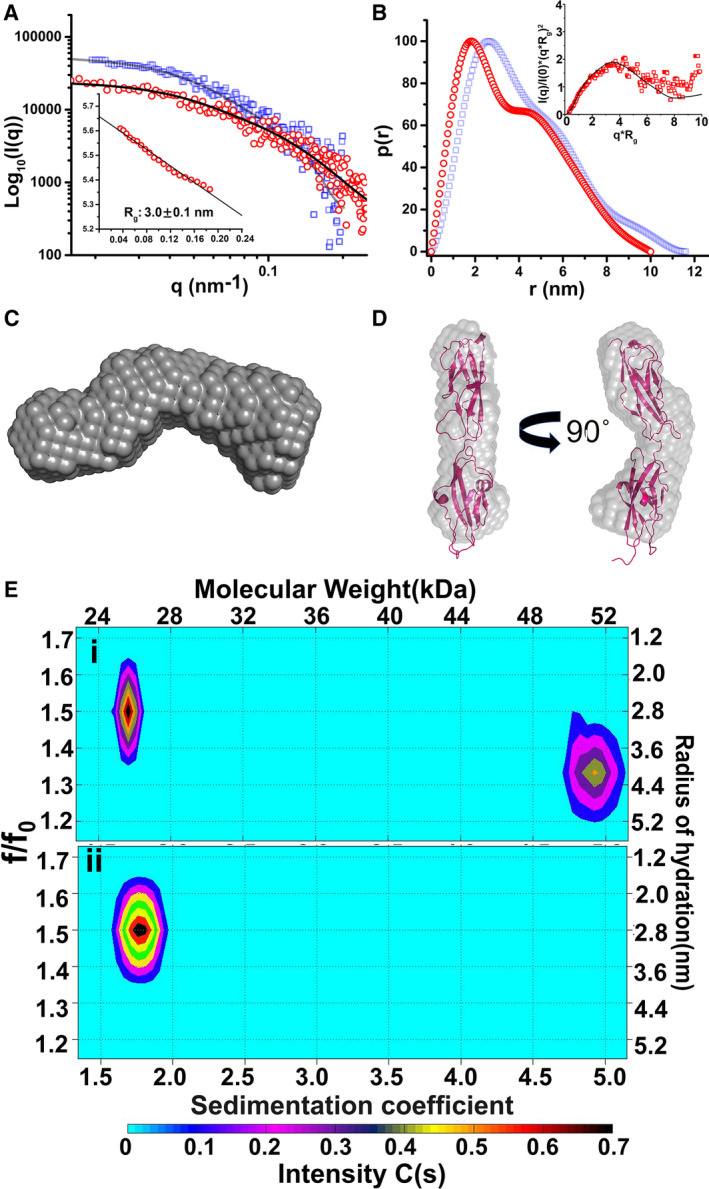
E78K mutant of Cdh23 EC1‐2 does not interact to form *trans*‐homodimer. (A) SAXS profile of the Cdh23 EC1‐2 mutant E78K is plotted as intensity I(q) vs. scattering vector (q) on a log–log scale. The solid black line represents the theoretical SAXS profile computed from the crystal structure. The Guinier plot (ln(*I*(*q*)) vs. *q* ^2^, *q* in nm^−1^) for the low q region is shown in the inset. The average scattering intensity was collected by exposing the sample to radiation for 20 min in 3 cycles. The acquisition was performed for 5 different batches of proteins, which makes *N* = 15. (B) The *p*(*r*) distribution for the E78K shows the maximum linear dimensions (*D*_max_) to be 10.0 nm. The normalized Kratky plot (*I*(*q*)/*I*(0)*(q*R_g_)^2^ of the experimental SAXS profile superimposed with that of the theoretical SAXS profile of the crystal structure is shown in the inset. (C) The average *ab initio* model obtained from DAMMIN is shown. (D) For a visual guide, the averaged model is fit to the Cdh23 EC1‐2 WT monomer (PDB ID: 2WHV) using SUPCOMB. (E) (i) The SV profile of Cdh23 EC1‐2 is presented with a color map to show the correlations between the sedimentation coefficients (s), frictional coefficients (*f*/*f*_0_), radii of hydration (*R*_H_), and molecular weights (MW) of the populations. Two populations in the profile confirmed the presence of dimer and monomer in solution when running at a concentration of 36 µm at 20 °C. (ii) The SV profile of Cdh23 EC1‐2 E78K at 30 µm showed a single population confirming the presence of monomer and is presented in color map to show the correlations between the sedimentation coefficients (*s*), frictional coefficients (*f*/*f*_0_), *R*_H_, and molecular weight.

We next performed molecular dynamics (MD) simulations of the rank 1 for 100 ns using GROMACS and delineated residue‐wise interactions (Materials and methods, Table S5). Stability of the dimer was achieved within 2 ns of the simulations to an average root mean square deviation (RMSD) of 0.34 nm (Fig. 4A,B). To identify the residues that are involved in dimerization, we compared the root mean square fluctuations (RMSF) of all residues between the monomer and dimer anticipating that residues buried within interacting surfaces may fluctuate less (Fig. 4C). The residues other than glycine that showed significant fluctuation differences are highlighted (Fig. 4C, inset). The time trace analysis of the MD results also indicated these residues responsible for the interactions. We divided these residues into two main interacting interfaces based on their positions in the EC domains (Fig. 4E–J). Interface one is dominated by electrostatic interactions that are mediated by the antiparallel overlap of strand F of the EC1 repeats. The residues involved in the interactions are K76, S77, E78, N97, and Q99, which are conserved in human, mouse, rat, and zebrafish (Fig. 5). Interestingly, S77 was found mutated into L(leucine) in patients who have cutaneous cancer. Some of these residues are also instrumental in heterodimerization with Pcdh15 at tip links. The other interface, which is amphiphilic, is formed by anchoring of the elongated N‐terminal strand of EC1 of one monomer to the β‐strand (G‐strand) of EC2 of the other protein. None of these residues are occluded by glycosylation, indicating that the interfaces are physiologically relevant (Fig. 6A). *In vitro* cell‐binding assays also showed the arrest of A549 cell lines on surfaces coated with Cdh23 EC1‐2 wild‐type (WT) with no post‐translational modifications. To understand the stability of the interface, we introduced a single point mutation at E78 to a positively charged residue, K (E78K). Proper folding of the mutant was verified using SEC and circular dichroism (Fig. 6B,C). To check the formation of the homodimer with mutants on a surface, we performed a live‐cell binding assay using A549 cells incubated on a glass‐surface 96‐well plate premodified with Cdh23 EC1‐2 (E78K) as described before (Materials and methods). We observed a significant decrease (78% drop) in the number of cells adhered to Cdh23 EC1‐2 (E78K) modified surfaces than WT (Fig. 6D). Quantitative estimation using MTT assay also measured a 35.8% decrease in the total number of viable cells bound to surfaces than Cdh23 EC1‐2 (WT)‐coated surfaces, suggesting the lack of homophilic interactions for the mutant (Fig. 2E). Similarly, *w*e observed the complete disruption of the dimer structure for the E78K mutant in solution even at a concentration of 10 mg·mL^−1^ (440 µm). The *R*_g_ of E78K obtained from SAXS is 3.0 ± 0.2 nm, corresponded to a monomer (Fig. 7A). The indirect Fourier transform gave a *D*_max_ of 10 nm (Fig. 7B). Further, we were able to fit the SAXS envelope (Fig. 7C) obtained for E78K to the crystal structure of the monomer (Fig. 7D). These values correlated well with the *R*_g_ and *D*_max_ of the monomeric WT protein (2.9 and 10.0 nm, respectively) as observed in the X‐ray crystal structure (PDB ID: 2WHV). Also, the molecular weight was estimated to be 23 kDa based on *V*_c_ and 30 kDa based on the scattering intensity of lysozyme. The deviation from the expected mass while using lysozyme as standard might be arising from an error in concentration. More importantly, both the methods estimated the molecular weight of Cdh23 EC1‐2 (E78K) to nearly half of the native, Cdh23 EC1‐2 dimer. We were able to fit the SAXS envelopes (Fig. 7C) obtained for E78K to the crystal structure of the monomer and the docked structure by aligning their inertial axis using SUPCOMB (Fig. 7D).

### The *trans*‐homodimer of Cdh23 EC1‐2 has a high dissociation constant, however, is long‐lived

**Figure 8:**
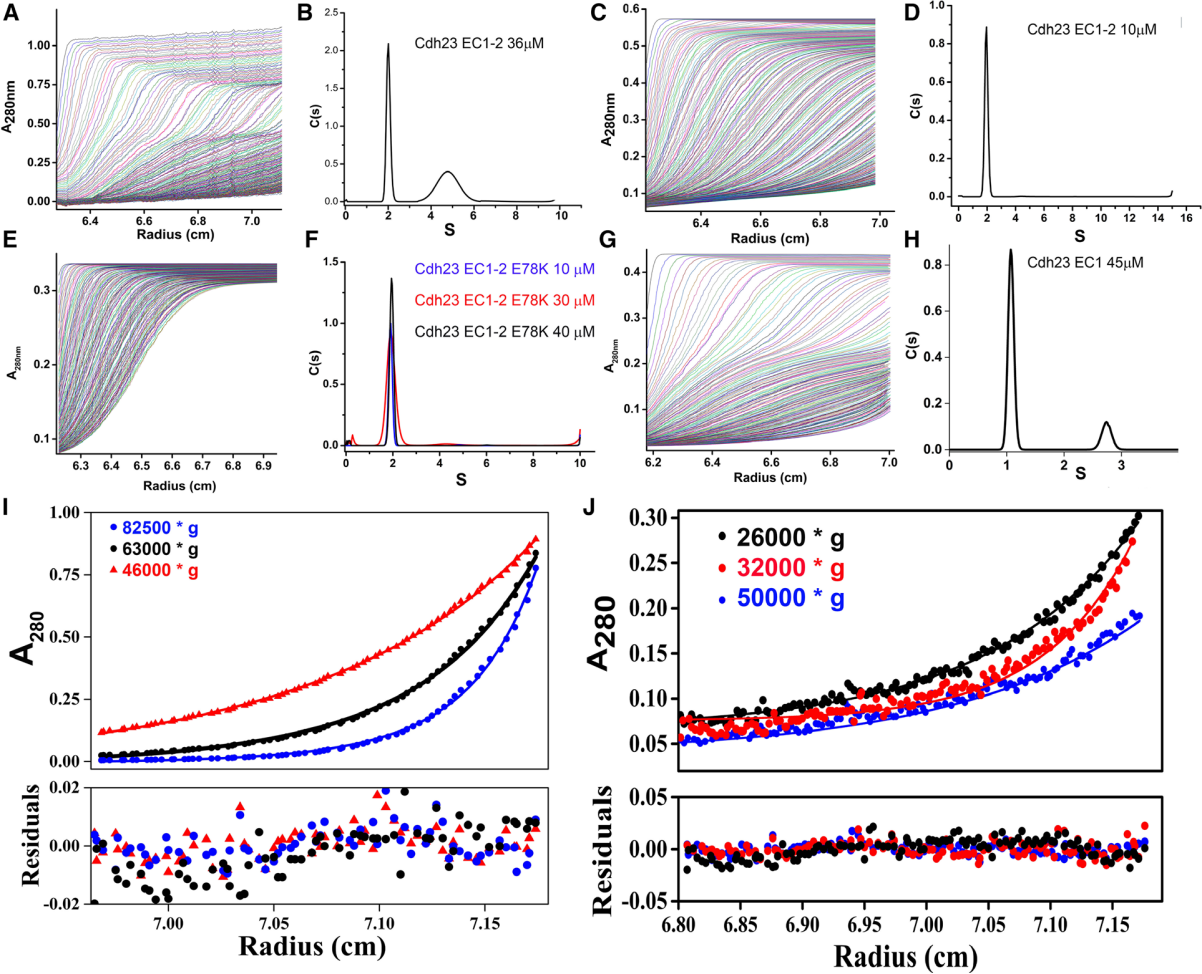
Quantitative estimation of the dissociation constants from the SV and SE run of Cdh23 EC1, Cdh23 EC1‐2 (WT), and Cdh23 EC1‐2 (E78K) mutant. Change in the absorbance at 280 nm with time across the radius of the cell is shown for (A) Cdh23 EC1‐2 at 36μM, (C) Cdh23 EC1‐2 (WT) at 10 μm, and (E) Cdh23 EC1‐2 (E78K) at 30 μm, and (G) Cdh23 EC1 WT at 45 µm. Figures B, D, F, and H present the corresponding SV profile (C(S) vs S) of Cdh23 EC1‐2 WT at 36 µm, Cdh23 EC1‐2 WT at 10 µm, Cdh23 EC1‐2 E78K at 30 µm, and Cdh23 EC1 WT at 45 µm, respectively. (I) SE experiments of Cdh23 EC1‐2 were run for 36 µm protein at three rotor speeds, 24 000 (red dots), 28 000 (black dots), and 32 000 rpm (blue dots). The solid lines represent the global fitting of the data to the monomer–dimer equilibrium model using SEDPHAT. The residuals of each fit are displayed below. (J) SE experiments of Cdh23 EC1 at 45 µm concentration for three different rotor speeds of 25 000 rpm (black circles), 20 000 rpm (red circles), and 18 000 rpm (blue circles). The data were monitored as absorbance at 280 nm. Solid lines represent the fitting to monomer–dimer equilibrium model using SEDPHAT software. *K*_D_ obtained for Cdh23 EC1 dimer was 52 ± 7 µm. We repeated all the experiments of both SV and SE for Cdh23 EC1‐2 WT and E78K proteins twice, which makes *N* = 2.

Once we deciphered the molecular structure of the homodimer of Cdh23, we were interested to measure the kinetic and thermodynamic parameters of the complex. We performed sedimentation velocity (SV) experiments with Cdh23 EC1‐2 (WT) (Fig. 7E,I) using analytical ultracentrifugation (AUC) at two protein concentrations, 10 µm, and 36 µm (Fig. 8A–D), with a rotor speed of 142 000 *g* at 20 °C. The SV run at the higher concentration produced two distributions of sedimentation coefficient (*C*(s)). The peak that appeared at 2.1 s corresponds to the monomer, while the peak at 4.77 s with a lower frictional coefficient ratio (*f*/*f*_0_) of 1.34 corresponds to the dimer (Fig. 7E(i)). Further, the dimer was confirmed from the estimation of the molecular weight (52 kDa) and *R*_H_ (4.1 ± 0.3 nm). The similar SV run for Cdh23 EC1‐2 (E78K) (Figs 7E(ii) and 8E,F), however, featured only single distribution for a monomer even at a concentration of ~ 440 µM (Fig. 7E(ii)). The *R*_H_ estimated for Cdh23 EC1‐2 (E78K) was 2.9 ± 0.1 nm (Fig. 7E(ii)).

We then performed sedimentation equilibrium (SE) using AUC with 40 µm of Cdh23 EC1‐2 (WT) protein (and 60 µm) at three different rotational speeds (46 000, 63 000, and 82 000 *g*) and estimated the *K*_D_ of monomer–dimer equilibrium as 18 ± 4 µm from the global fitting of the equilibrium curves using free software sedphat (Fig. 8I) 29. The *K_D_* obtained for Cdh23 EC1‐2 (WT) is significantly lower than E‐cadherin (96.5 ± 10.6 µm) 30, however, comparable with N‐cadherin (25.8 ± 1.5 µm) 30 and R‐cadherin (13.7 ± 0.2 µm) 31. Interestingly, we observed only one dominating peak with a C(s) of 1.96 s and a *f*/*f*0 of 1.51 corresponding to a monomer when we ran SV with 10 µm for Cdh23 EC1‐2 (WT) (Fig. 8C,D), even though the dimer‐to‐monomer ratio at 10 µm of protein is nearly 1/4^th^ for a homodimer with *K*_D_ of 18 ± 4 µm. No detection of the dimer here could be an artifact from the detection limit of the instrument or could be due to slow binding on‐rate of Cdh23 EC1‐2 (WT) toward dimerization 32. Moreover, it is well documented that the *K*_D_ obtained from SV or SE varies widely for the same proteins and these variations are mainly attributed to the differences in internal pressure in AUC cell arising from different run‐time 33, 34, 35, 36.

Since the interface one is mediated by EC1 alone, we checked whether the interface one interacts independently to form the homodimer. As expected, SV with Cdh23 EC1 (WT) showed a population of both monomers and dimers indicating that EC1 alone can form a homodimer with a lower affinity (*K*_D_ ~ 52 ± 7 µm, Fig. 8G,H,J) than EC1‐2. Interestingly, we did not observe any dimer in SEC when injected 100 µm of Cdh23 EC1 which should contain 80% of the dimer. The nonappearance of the dimer peak in SEC could be due to in‐column dilution of the protein before elution.

**Figure 9:**
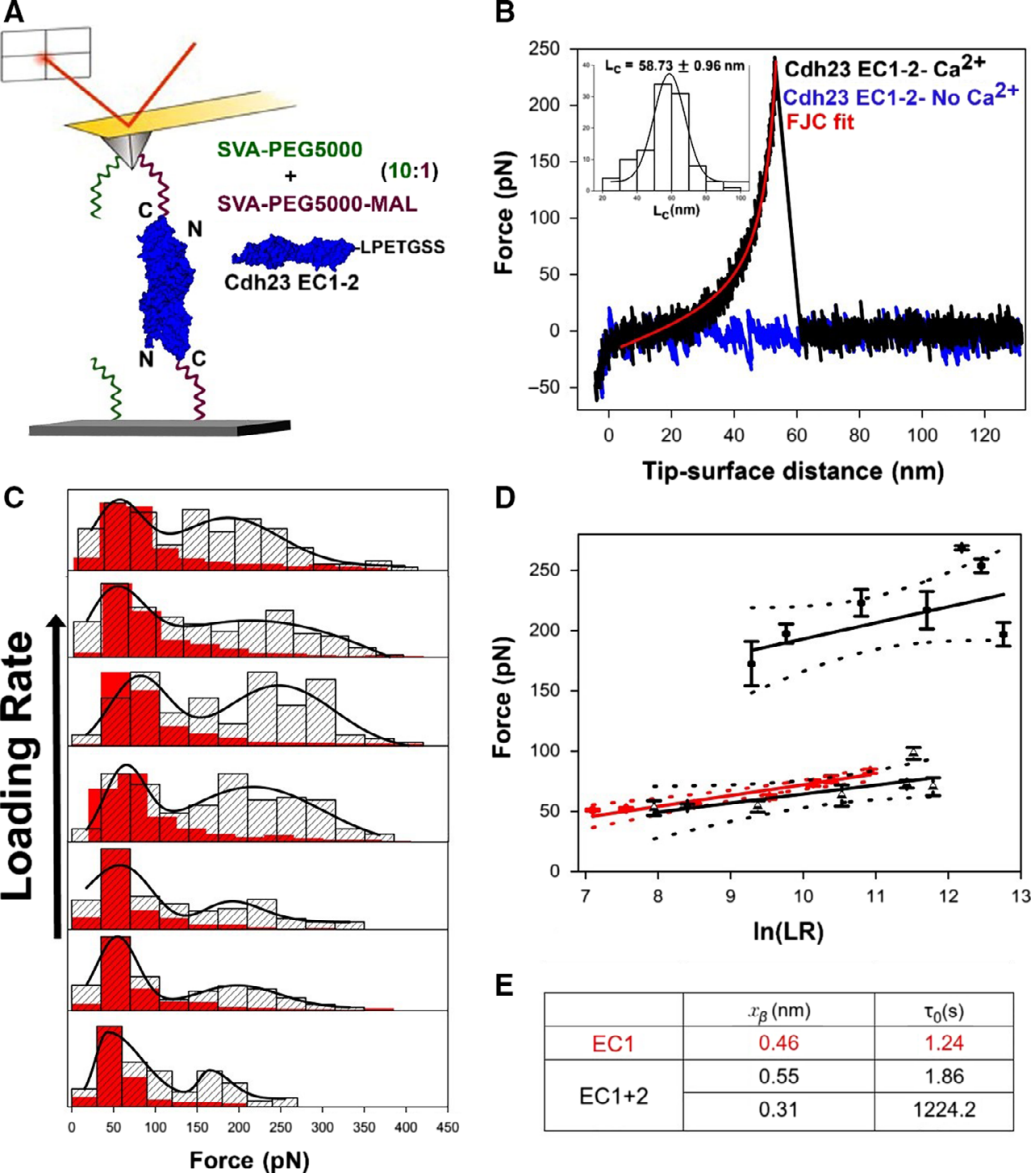
SMFS of Cdh23 EC1‐2 dimers reveals two binding conformations. (A) Schematics show the AFM cantilever and coverslip functionalized with proteins for the single‐molecule dynamic force spectroscopy experiments. The C terminus of the protein is covalently attached to the cantilever and coverslip using sortase enzyme chemistry. A mixture of monofunctional and bifunctional polyethylene glycol (PEG, 5 kDa) is used as a spacer to minimize nonspecific and multiple events. (B) A typical single‐molecule unbinding force curve featuring a characteristic stretching of PEG is shown in the black solid line. The fit to the freely jointed chain (FJC) model is shown in red. The rupture force was estimated from the peak maximum and contour length (*L*_c_) from the FJC fit. The inset shows a Gaussian distribution for *L*_c_ for the measurements performed at a 13841 pN/s loading rate. The mean *L*_c_ was obtained from the Gaussian fit (solid line). The blue line represents no unbinding events. (C) The histograms of unbinding forces with increasing loading rates show a bi‐Gaussian distribution for Cdh23 EC1‐2 (gray) and uni‐Gaussian for Cdh23 EC1 (red). Solid black curves were generated from the bi‐Gaussian fitting of the distribution and used as a visual guide. From the peak maxima, we obtain the two most likely forces (*F*_mp_) for Cdh23 EC1‐2 and one for Cdh23 EC1 at each loading rate. (D) The dependence of *F*_mp_ on ln(loading rate) is shown for Cdh23 EC1‐2 (black circles for the high‐force regime and black triangles for the low‐force regime) and Cdh23 EC1 (red circles). The error bars represent the SEM estimated using data from five different experiments (*N* = 5). Each set of data is fitted to a Bell–Evans model (superimposed solid lines). Dotted lines indicate the 95% confidence intervals of the data. (E) The table summarizes the parameters, *x*_β_ (maximum distance to unbinding in the reaction coordinate) and *τ*_0_ (lifetime at zero force) for EC1 and EC1‐2 obtained from the Bell–Evans model fit.

Finally, we performed SMFS with Cdh23 EC1‐2 (WT) to estimate the strength of the interfaces as *in cellulo* studies measured strong adhesive properties of Cdh23 that suppresses tumor metastasis. For SMFS, we covalently attached the C terminus of the protein to AFM cantilevers (Si_3_N_4_) and glass coverslips using the sortagging protocol 37 (Fig. 9A, Materials and methods). A typical single‐molecule unbinding force curve (unbinding event, black line) and a no‐event (blue) curve that is obtained for SMFS are shown in Fig. 9B. Overall, we observed 8–10% unbinding events, 97% of which featured single unbinding force curves that fit the freely jointed chain (FJC) model (Fig. 9B). These data corroborate well with the Poisson statistics used for single‐molecule sorting. We plotted the maximum unbinding forces for seven different loading rates as histograms (Fig. 9C). The contour length (*L*_c_) was estimated as an FJC fitting parameter for each of the individual unbinding curves, which distributed as a single Gaussian centered at 58.7 ± 1.0 nm irrespective of the pulling rate (Fig. 9B, inset). This finding is typical for the stretching of two PEG molecules of 5 kDa molecular weight in series. The unbinding force distributions for Cdh23 EC1‐2 (WT), however, showed two well‐separated binomial distributions (Fig. 9C). We hypothesized that the two distributions correspond to two different binding states: The low‐force distribution is due to fast binding by either of the two interacting interfaces, and the high‐force distribution is due to a complete handshake binding between Cdh23 EC1‐2 (WT) partners. For verification, we repeated the force spectroscopy with Cdh23‐EC1(WT) alone and measured the strength of interface one. As confirmation, the unbinding force distribution obtained for EC1 alone (red, Fig. 9C) perfectly overlaid with the low‐force distribution of Cdh23 EC1‐2. We also measured a higher probability of events for Cdh23‐EC1 (WT) (~10%) than for EC1‐2 (WT) (~6%). This may refer to a higher binding on‐rate of Cdh23‐EC1 (WT) than Cdh23 EC1‐2 (WT).

We then plotted the most probable forces (*F*_mp_) obtained from the peak maxima of the distributions with increasing loading rates. The loading rate for each velocity was estimated by considering the molecular tether and cantilever in series (Materials and methods) 38, 39. The data were fitted to the Bell–Evans model (Eq. 6) 40, 41, and the intrinsic lifetime (*τ*_0_) and the minimum distance from the bound state to the transition state (*x*_β_) were determined (Fig. 9D,E). For Cdh23 EC1‐2 (WT), we measured two *F*_mp_ values for two force distributions at each loading rate. Our results indicate a very high *τ*_0_ of 1224.2 s for Cdh23 EC1‐2 (WT) for the high‐force distribution and *x*_β_ of 0.31 nm. The *τ*_0_ and *x*_β_ obtained for the low‐force distributions are 1.86 s and 0.55 nm, respectively, which corresponded to the values obtained for Cdh23‐EC1 (WT) alone with a 95% confidence interval, corroborating with the SV run. Next, we repeated the force spectroscopy experiments with Cdh23 EC1‐2 (E78K) mutant and measured very low frequency of events similar to nonspecific measurements confirming again that Cdh23 EC1‐2 (E78K) mutant does not interact with each other.

## Discussion

Cdh23 mediates stronger cell–cell adhesion via homophilic trans‐binding than classical E‐cadherin 10. The implication of such strong adhesion by Cdh23 in physiology is metastatic suppression 6. It, therefore, became imperative to decipher the structural basis of the strong adhesion. From a barrage of biophysical and biochemical data, here we presented the first view of the homophilic trans‐binding structure of Cdh23. The binding interface is mediated by the extended antiparallel overlap of the two terminal domains covering a surface area of 1796.3 Ǻ^2^ per monomer. Experiments including analytical SEC at 4 °C, smFRET at 20 °C, AUC at 20 °C, SAXS at 20 °C, DLS at 20 °C were performed to verify and model the homodimer. Irrespective of the methods and their working temperatures, the physical parameters like molecular weight (MW), the radius of gyration (*R*_g_), the radius of hydration (R_H_), estimated from the different methods have all converged (Table S6). Even the geometry of the dimer estimated from the *R*_g_/*R*_H_ values from different techniques predicted a nonspherical geometry, as obtained from the SAXS (Table S7). Molecular details from in sillico MD studies further revealed two distinct interfaces in the homodimer: electrostatic interactions between EC1 and EC1 and hydrophobic interactions between EC1 and EC2 of the opponent partners. The extended overlap of domains may impose stronger adhesion as observed in cellulo than the single domain overlap in classical cadherins. In classical cadherins, the thermodynamically stable trans‐dimer is formed by the exchange of the N‐terminal β‐strands of the distal domain alone.

To quantify the adhesive strength of Cdh23 homodimers, we measured the lifetime (*τ*_0_ = 1/*k*_off_) of the complex from SMFS experiments using AFM. Two binding conformations were identified: one with interface one or EC1 alone, at a lower force range but with higher binding probability, and the other at a higher force range comprising both interfaces. The strength for interface one was weak (*τ*_0_ = 1.86 s) and comparable to that of classical cadherins (Table S8). The strength of the final structure (*τ*_0_ = 1224 s) is among the strong interactions between cadherins. The longer lifetime of the trans‐dimer of Cdh23 was expected from the extended binding interface. The long lifetime of the Cdh23 homodimer thus clarifies the strong aggregation index measured for cells expressing Cdh23 than the cells adhered via classical E‐cadherin. However, the *K*_D_ measured for Cdh23 is in the micromolar range, comparable to the classical cadherins (Table S8). The similar trend was also observed for nonclustered protocadherins (protocadherin‐19, for example), which too forms homodimers with extended overlap between multiple EC domains with *K*_D_ in micromolar ranges 2. The disparity in the *K*_D_ values with the area of the interacting interface may be attributed to the slow on‐rate (~10^6^ M^−1^s^−1^) of the interactions which can be computed from the ratio of the off‐rate and *K*_D_.

The interface of Cdh23 mediated trans‐homodimer is amphiphilic and involves residues that are conserved across a variety of species (Fig. S2). A missense mutation at the binding interface was identified in patients who have cutaneous cancer, indicating a physiological relevance of the interface. Further, the interface was validated by introducing a single point mutation (E78K), which impaired the dimer complex.

Deciphering the contribution of nonclassical cadherins in cell adhesion is gaining interests, especially the giant cadherins with long extracellular domains 42, 43, 44. Primary focus on this area has been to identify nonclassical cadherin mediating cell adhesion junctions 45, 46, 47, understand their packing conformations at the junction 42, 48, the function of the junction 44, 49, 50, and the molecular structure of the junction 8. Deciphering the molecular structure of the trans‐homodimer of Cdh23 is an essential and timely observation in this context, which may pave the way for understanding the molecular details of the strong cell adhesion junctions by atypical cadherins.

## Materials and methods

### Cloning, expression, and purification

Mouse Cdh23 (NP_075859.2) extracellular (EC) repeats EC1 (Q24 to D124) and EC1‐2 (Q24 to D228) were PCR‐amplified and cloned into pET21a vector (Novagen, Merck‐Sigma, St. Louis, MO, USA). We considered the Q24 as Q2 in our experiments. Cdh23‐EC1, EC1‐2, and EC1‐3 repeats were expressed in *E. coli* BL21CodonPlus (DE3)‐RIPL cells (Stratagene, San Diego, CA, USA) and *E. coli* lemo(DE3) cultured in Luria/Bertani broth (Hi‐Media, Mumbai, Maharashtra, India) and grown at 37 °C to an OD_600_ of 0.6. Induction was carried out differently for both the strains. For expression in *E. coli* lemo(DE3), the cells were induced along with 1 mm rhamnose and 0.2 mm IPTG (Hi‐Media) and then induced at 30 °C for 6 h. For expression in *E. coli* BL21CodonPlus (DE3)‐RIPL, the protein was then induced with 0.2 mm IPTG and incubated at the same temperature. Unlike in *E. coli* BL21CodonPlus (DE3)‐RIPL, Cdh23 EC1‐2 protein from *E. coli* lemo(DE3) was obtained in the soluble fraction. For expressions in *E. coli* BL21CodonPlus (DE3)‐RIPL, we revived the proteins from the inclusion bodies and processed through serial dialysis, as reported 8. All proteins were first purified using Ni‐NTA beads followed by SEC on a Superdex 200 column (GE Healthcare, Chicago, IL, USA) in 25 mm HEPES (pH 7.5), 25 mm KCl, 100 mm NaCl, and 2 mm CaCl_2_.

### Analytical size exclusion chromatography

We injected 100 μm of purified Cdh23 EC constructs on Superdex 200 increase 10/300 column (GE Healthcare) at a flow rate of 0.3 mL·min^−1^. Prior to the loading of the protein, the column was washed with degassed ultrapure water and equilibrated with degassed SEC buffer (25 mm HEPES, 25 mm KCl, 50 mm NaCl, 2 mm CaCl_2_, pH 7.5). The column was calibrated using standard protein mix kit (Merck‐Sigma) with proteins, cytochrome‐C (12 000 Da), carbonic anhydrase (29 000 Da), albumin (66 000 Da), and alcohol dehydrogenase (76 000 Da). The standard calibration curve was generated by estimating the *K*_av_ (partition coefficient) using the equation, $\frac{V_{e}-V_{0}}{V_{c}-V_{0}}$, where *V*_e_ is the elution volume (*V*_e_), *V*_0_ is void volume, 8 mL for a 24 mL (*V*_c_) Sephadex 200 Increase size exclusion column. The eluted fractions for all the proteins were run on SDS/PAGE for molecular weight confirmation.

### Single‐molecule FRET measurements

For N‐terminal labeling with dyes, we recombinantly modified valine at position 3 to cysteine (V3C) and attached to maleimide dyes (supplied by Lumiprobe) using the thiol–maleimide Michael addition reaction. The unreacted dye was removed using spin columns (10 kDa MWCO). The labeling ratio was measured from the absorbance at 280 nm for protein, 545 nm for Cy3, and 645 nm for Cy5. For C‐terminal labeling, we followed sortase A (srtA)‐mediated enzymatic reaction. For sortagging, we recombinantly modified the C‐terminal of the proteins with ‐LPETGGS. SrtA recognizes the sequence and inserts polyglycine by cleaving the T‐G peptide bond. To introduce the dye, we used dye‐modified polyglycine (GGGC‐dye) 31.

smFRET measurements were performed using IX83 P2ZF inverted microscope (Olympus, Shinjuku, Tokyo, Japan) combined with IX3 TIR MITICO TIRF illuminator equipped with 532 nm diode laser system for cy3 excitation and 645 nm diode laser system for cy5 excitation. Fluorescence was collected using an oil‐immersion objective (60X, NA 1.45, Olympus) into an EMCCD camera (Q‐Imaging Roller Thunder, Surrey, BC, Canada). Image acquisition and processing were performed using CellSens Dimension (Olympus) software. iSMS software was used to localize the single‐molecule dye pairs on the surface and then analyzed for their intensity profiles. The background subtraction and drift correction were also done using the same software. The FRET efficiency for each pair of donor–acceptor proteins was estimated using the same software. From the efficiency distributions, we estimated the distance between the FRET pairs using the following equation,

(1) $$E=\frac{1}{1+{\left(\frac{R}{R_{0}}\right)}^{6}}$$

For smFRET on surfaces, glass coverslips were freshly cleaned, silanized, pegylated, and finally modified with C‐terminal of proteins specifically following a sortagging protocol as described elsewhere 31.

### Live‐cell binding and cell–cell aggregation assays

For the live‐cell binding to surfaces, glass‐bottom 96‐well plates were cleaned, silanized, pegylated, and modified with proteins specifically. The C‐terminal of the protein was attached covalently to the surfaces using sortagging as described before. Protein‐coated surfaces were incubated with live A549 cells (10^4^ cells) per well in 2 mm Ca^2+^ buffer. After an incubation of 2 h, the surfaces were gently washed twice for 5 min with HEPES‐Ca^2+^ buffer and imaged for assessing cell density. The Ca^2+^ from the surface was chelated by incubating with 1 mm EGTA, and again, the cell density was monitored. To number of live cells adhered to surfaces was quantified using MTT reagent. MTT reagent was added to each well and incubated for an hour. The cells were then lysed with DMSO, and the OD was monitored at 570 nm.

For cell–cell aggregation assay, the cells 36 h after the transfection with Cdh23 were counted and resuspended in HBSS buffer supplemented with Ca^2+^ ions to a final cell count of ~ 10^5^ cells. The aggregation was initiated by incubating the cells at 80 rpm and then imaged using bright field at 10× magnification using a Leica microscope.

### SAXS data acquisition and analysis

The SAXS data were acquired for a q‐range of 0.1 to 4 Å^−1^ on a SAXSpace instrument (Anton Paar GmbH). The X‐ray scattering setup had a slit‐collimated X‐ray source with a wavelength of 0.154 nm. The data were collected on a Mythen (Dectris, Baden‐Daettwil, Switzerland) detector placed at a distance of 317.6 mm from the sample for 60 min (20 min × 3 frames). SAXStreat software was used to calibrate the data for the beam position. The saxsquant software was then used to subtract buffer contribution, set the usable q‐range, and desmear the data using the beam profile. For each experiment, 100 µL of protein solution (Cdh23 EC1‐2 and its mutant E78K) and their corresponding buffers were exposed to X‐rays in a quartz capillary at a temperature of 10 °C. Data processing provided the scattering intensity (*I*) as a function of momentum transfer vector *q* (*q* = 4πsinθ/λ, where θ and λ are the scattering angle and the X‐ray wavelength, respectively). The normalized Kratky plots (*I*(*q*) × *q* ^2^ (*q* × *R*_g_)^2^/I(0) vs. *q* × *R*_g_)) were made from the SAXS data using the program scatter (http://www.bioisis.net/) to interpret whether the protein remains folded during the SAXS data collection. The Guinier approximation was carried out using primusqt 24 of atsas 2.7 suite of programs 51 to estimate the radius of gyration (*R*_g_) of the major scattering species.

Using gnom program 26, we carried out the indirect Fourier transformation of SAXS data to obtain the probability distribution of the pairwise vectors (*P*(*r*) curve) arising from scattering of the protein molecule in solution. The *P*(*r*) curve analysis was done to acquire the maximum linear dimension (*D*_max_) and the *R*_g_ in real space.

### Shape reconstruction

Ten independent models were generated using dammif 27 program. The models were aligned, averaged, and filtered using damaver 52 suite of programs. The averaged envelope was further refined using dammin 28 program. This procedure provided an envelope that reflected the shape of a protein molecule in solution.

### Protein docking

PatchDock is an online tool that follows rigid‐body docking optimization with shape complementarity and two‐point interactions between hot‐spots. Hot‐spots are decided based on residues that are conserved in protein–protein interaction surfaces and mediate salt‐bridge type interactions, H‐bonding, hydrophobic interactions, aromatic pi‐stacking etc.

The available crystal structure of monomer Cdh23 EC1‐2 was docked using PatchDock server 53 to generate the homodimer. *Z*‐test was performed using SAXS‐based parameters like linear dimension and *R*_g_ and FRET‐based end‐to‐end distances between the docked models and SAXS‐based envelop (Table S7). The structures which agreed the most with the SAXS‐based envelope of the protein were overlaid by computationally aligning using supcomb 54 program. Program pymol 55 was used for graphical analysis and figure generation.

### Steady‐state fluorescence and time‐resolved anisotropy experiments

The fluorescence properties of Trp in proteins were probed by exciting at 295 nm and monitoring the emission for 310–450 nm (λ_em_ = 338 nm) at 25 °C using Jobin Yvon Fluoromax‐4 spectrofluorometer equipped with a PMT detector. The slit width (2 nm), step size (0.1 nm), and integration time (0.05 s) were maintained for all experiments.

Fluorescence anisotropy decay measurements were performed using TCSPC (Fluorocube, Horiba Jobin Yvon, Kyoto, Japan). For decay measurements, the emission polarizer was set to 0° with respect to excitation polarizer for parallel measurements and at 90° for perpendicular measurements. A 293 nm laser diode was used as an excitation source, and the emission monochromator for tryptophan was fixed at 342 nm, at a slit width of 8 nm. The instrument response function was measured using 2% LUDOX (Sigma‐Aldrich).

The anisotropy decay was calculated using Equation (2) and was fitted to biexponential decay fit to determine the values of rotational correlation times using Equation (3). The values are tabulated in Table S6.

(2) $$r\left(t\right)=\frac{I_{⊥}\left(t\right)-I_{\|}\left(t\right)}{I_{⊥}\left(t\right)+2I_{\|}\left(t\right)}$$

(3) $$r_{t}=r_{0}\left[A_{1}e^{\frac{-t}{\phi 1}}+A_{2}e^{\frac{-t}{\phi 2}}\right]$$

where *I*_⊥_ is the vertical emission and *I*_‖_ is the horizontal emission.

### MD simulations

MD simulations were performed on the in‐house workstation. The crystal structure of the docked model which agreed the most with the SAXS‐based envelop was used for simulations. Simulations were performed with GROMACS 5.0.1 using All‐atom OPLS force field and TIP4P water model. All analyses were performed using VMD. For MD simulations, the Cdh23‐dimer model was placed at the center of a 13.6 × 5.7 × 5.8 nm triclinic box filled with four‐point charge water molecules such that no atom of the protein was closer than 1 nm from the walls of the box. The MD equilibration system consisted at an average of 54 435 atoms. System charge neutrality of the system was maintained by adding Na^+^ counterions to the box as needed with buffer ions (50 mm NaCl, 50 mm KCl, and 2 mm CaCl_2_). To ensure that the solvated Cdh23‐dimer model has no steric clashes or inappropriate geometries, we first energy minimized the system. Periodic boundary conditions were assumed in all simulations. A cutoff of 1 nm was used for *van der Waals* interactions. Electrostatic interactions were calculated with a particle mesh technique for Ewald summations with a 1 nm cutoff. We equilibrated the water molecules and ions around the Cdh23‐dimer model in two phases. In the canonical (NVT) phase, the system was established at a constant reference temperature of 300 K. The pressure of the system was then stabilized under isothermal‐isobaric (NPT) conditions. Following equilibration, 100 ns MD simulations were run at 2 fs integration steps and frames were recorded at 1‐ps interval. We repeated the simulations 3 times with the same docked structure and obtained overlapping results. We used visual molecular dynamics (VMD) for H‐bond analysis.

### Analytical ultracentrifugation

Analytical ultracentrifugation (AUC) experiments were carried out using a Beckman XLA/I ultracentrifuge and equipped with a Ti50An rotor using 12‐mm six‐channel cell centerpieces with sapphire windows and detection by UV at 280 nm. Both sedimentation velocity and equilibrium experiments were performed at 20 °C at pH 7.6 buffer containing 25 mm HEPES, 50 mm NaCl, 25 mm KCl, and 2 mm CaCl_2_. Prior to each experiment, the protein sample was dialyzed in the buffer for 18 h. Sedimentation velocity experiments were performed for all proteins at a rotor speed of 142 000 *g*. 300 scans were taken consecutively. Data were analyzed using sedfit software following continuous C(s) distribution, C(M) distribution, and C(s), (*f/f_0_*) models. For equilibrium, samples were subjected to fast spins at 99 000 *g* for 20 h to achieve rapid equilibrium. Then, we took one scan after each one‐hour interval for 5 h and checked the rmsd fluctuations to test whether samples had reached equilibrium or not. Then, we decreased the rotor speed to 32 000 rpm and wait for 4 h before taking 3 scans consecutively. The same procedure has been followed for two other speeds 28 000 and 24 000 rpm, respectively. Buffer viscosity and density were measured using SEDNTERP (http://sednterp.unh.edu/). SEDPHAT was used to estimate the dissociation constant. We performed global fitting with mass conservation following monomer–dimer association model keeping baseline, meniscus, bottom, binding affinity as floating parameters.

### SMFS experiments

For SMFS using AFM (Nano Wizard 3, JPK Instruments, Berlin, Germany), the protein molecules were immobilized on the glass coverslip and Si_3_N_4_ cantilever (Olympus, OMCL‐TR400PSA‐1), using a high‐specificity immobilization protocol as described before 31.

For quantitative estimation of force from each experiment, the spring constant of the cantilever was measured from the thermal noise using thermal fluctuation methods 56. For dynamic force spectroscopy, numerous force–distance curves were recorded at different pulling rates (500, 750, 2000, 5000, 7500, 10 000, and 15 000 nm/s) while keeping approach and retract distance of 200 nm at 6 kHz sampling rate and a contact time of 500 ms constant. A total of around 6000 curves were recorded at each velocity.

The analyses for the plotted force curves were done in MATLAB with home‐written programs. The single‐molecule events were selected from the fit to freely joint chain (FJC) model using the following equation:

(4) $$l\left(F\right)=L_{c}\left[\coth\left(\frac{\mathrm{Fa}}{k_{B}T}\right)-\left(\frac{k_{B}T}{\mathrm{Fa}}\right)\right]$$

where *α* is Kuhn length, *L*_c_ is contour length (CL), and *l*(*F*) is stretching of PEG at every force. The unbinding forces were estimated from the peak maxima of the single‐molecule unbinding events for each loading rate and plotted as distribution. Bin width was estimated from Scott’s method 57. Each force distribution was hence fitted to Gaussian distribution, and the most probable force (*F*) for each loading rate was obtained from the fit. Loading rate at each velocity was calculated using the following equation 39

(5) $$\frac{1}{v_{F}}=\frac{1}{k_{c}v}+\frac{1}{v}\frac{dl\left(F\right)}{dF}=\frac{1}{k_{c}v}\left[1+\frac{k_{c}L_{c}a}{k_{B}T}\left({\left(\frac{k_{B}T}{\mathrm{Fa}}\right)}^{2}-{\text{csch}}^{2}\left(\frac{\mathrm{Fa}}{k_{B}T}\right)\right)\right]$$

where *α* is Kuhn length, *v* is pulling velocity, *k*_c_ spring constant of the cantilever, *L*_c_ is the contour length, and *v_F_* is the loading rate.

The most probable force (*F*) with loading rate (*v_F_*) was fitted to Bell–Evans model 40, 41 and estimated the kinetic parameters like off‐rate (*k*_off_), transition distance (*x*_β_) using the equation:

(6) $$F\left(v_{F}\right)=\left(\frac{k_{B}T}{x_{\beta }}\right)\ln\frac{v_{F}x_{\beta }}{k_{\text{off}}k_{B}T}$$

Nonspecific binding rates were estimated in two different ways: by modifying either of the two surfaces (the cantilever or the coverslip) identically but without attaching proteins or by performing the same single‐molecule protein–protein unbinding experiments in the absence of Ca^2+^ ions in the Chelex buffer and EGTA buffer. In both cases, the nonspecific events accounted for < 0.5% of the total number of total selected PEG stretching events.

## Conflict of interests

The authors declare no conflict of interest.

## Author contribution

SR has supervised the project. GSS and JSS did the cloning, expression, and purification. GSS, AK, AS, A, and SR recorded and analyzed SAXS. JPH recorded and analyzed AUC. GSS, AK, AS, RMY, and SR analyzed MD. GSS performed SMFS, smFRET, cell aggregation, CD, SDS/PAGE, WB. MKS, and GSS performed live‐cell binding assay. GSS, AS, MKS, and JPH made the figures. SR and GSS wrote the manuscript. GSS, AK, JSS, JPH, AS, A, MKS RMY, and SR edited the manuscript. AS, AK, and JSS are co‐second authors.

## Supporting information


**Fig. S1.** Multiple sequence alignment showing key residues driving homodimerization in type I and type II cadherins versus Cdh23
**Table S1.** End to end distances obtained from smFRET data analysis along with photo‐physical properties of fluorophores.
**Table S2.** Data collection parameters are tabulated along with the software used for analysing the scattering data.
**Table S3.** Determination of the rotational correlation decay time of Trp66 (W66) at three different concentrations of Cdh23 EC1‐2 WT.
**Table S4.** Quantitative comparison of different Patch‐Dock structures.
**Table S5.** Parameters used during MD simulations.
**Table S6.** Physical parameters of the trans‐homodimer of Cdh23 EC1‐2 (WT), estimated from various techniques.
**Table S7.** Estimation of R_g_/R_H_ of the trans‐homodimer of Cdh23 EC1‐2 (WT) obtained from various methods.
**Table S8.** A comparison of dissociation constants and off‐rate values for first two domains of various cadherins.
