# Supplementary material for: Structural basis of the strong cell‐cell junction formed by cadherin‐23
Source: FEBS J. 2019 Dec 11;287(11):2328–47. doi: 10.1111/febs.15141 (PMC7317872; doi:10.1111/febs.15141)
Supplement: Supplementary file 1 — Fig. S1. Multiple sequence alignment showing key residues driving homodimerization in type I and type II cadherins versus Cdh23 Table S1. End to end distances obtained from smFRET data analysis along with photo‐physical properties of fluorophores. Table S2. Data collection parameters are tabulated along with the software used for analysing the scattering data. Table S3. Determination of the rotational correlation decay time of Trp66 (W66) at three different concentrations of Cdh23 EC1‐2 WT. Table S4. Quantitative comparison of different Patch‐Dock structures. Table S5. Parameters used during MD simulations. Table S6. Physical parameters of the trans‐homodimer of Cdh23 EC1‐2 (WT), estimated from various techniques. Table S7. Estimation of Rg/RH of the trans‐homodimer of Cdh23 EC1‐2 (WT) obtained from various methods. Table S8. A comparison of dissociation constants and off‐rate values for first two domains of various cadherins. [file FEBS-287-2328-s001.zip › febs15141-sup-0001-Suppinfo.pdf]

FEBS PRESS
science publishing by scientists
WILEY
The FEBS Journal
www.febsjournal.org

Structural basis of the strong cell-cell junction formed by cadherin-23

Gayathri S. Singaraju, Amin Sagar, Anuj Kumar, Jesse S. Samuel, Jagadish P. Hazra, Malay K. Sannigrahi, Ragothaman M. Yennamalli, Fnu Ashish and Sabyasachi Rakshit

DOI: 10.1111/febs.15141

Supplementary Information

Molecular mechanism of the cell-cell adhesion by atypical Cadherin-23

G. S. Singaraju, A. Sagar, A. Kumar, J. S. Samuel, J. P. Hazra, M. K. Sannigrahi, R. M. Yennamalli, FNU Ashish and S. Rakshit*

This PDF contains

Supplementary Figs 1

Supplementary Tables 1 to 8

Index

Figure 1: **Multiple sequence alignment showing key residues driving homodimerization in type I and type II cadherins versus Cdh23.**

Table 1: **End to end disances obtained from smFRET data analysis along with photo-physical properties of fluorophores.**

Table 2: **Data collection parameters are tabulated along with the software used for analyzing the scattering data.**

Table 3: **Determination of the rotational correlation decay time of Trp66 (W66) at three different concentrations of Cdh23 EC1-2 WT.**

Table 4: **Quantitative comparison of different Patch-Dock structures**

Table 5: **Parameters used during MD simulations.**

Table 6: **Physical parameters of the trans-homodimer of Cdh23 EC1-2 (WT), estimated from various techniques.**

Table 7: **Estimation of R_g/R_H of the trans-homodimer of Cdh23 EC1-2 (WT) obtained from various methods.**

Table 8: **A comparison of dissociation constants and off-rate values for first two domains of various cadherins.**

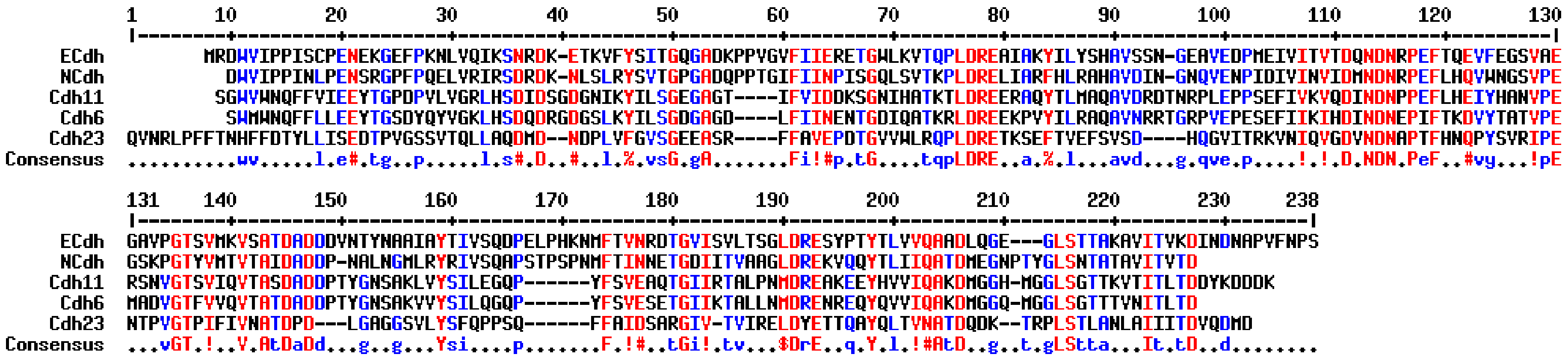

Supplementary Figure 1: Multiple sequence alignment showing key residues driving homodimerization in type I and type II cadherins versus Cdh23. Sequence alignment results obtained using an online tool, Kalign2 [1] show that the binding interfaces of type I, ECdh (UniProt accession no. P09803) and NCdh (UniProt accession no. P15116) and type II, Cdh6, (UniProt accession no. P97326) and Cdh11 (UniProt accession no. P55288) show that the residues conserved for dimer formation, W2 or W2 and W4 are not conserved in Cdh23 (UniProt accession no. Q99PF4), while the key residues that we identified for the dimer formation of the latter are conserved in the rest. This shows that dimer model for Cdh23 differs from those of the know models of the cadherin family.

Supplementary Table 1: End-end distances obtained from smFRET data analysis along with photo-physical properties of fluorophores.

Possible Dimer Conformation	Forster radius R_0 (nm)	E_{FRET}^{mp} $E_{FRET}^{mp} = {}^{1}\big/_{1 + ({}^{R}/_{R_0})^6}$	R (nm)
EC1-2 (N_DC_A)	5.4	0.59±0.002	5.0±0.08
EC1-2 (N_DN_A)	5.8	0.40±0.005	6.1±0.01
EC1-3 (N_DC_A)	5.4	0.42±0.004	6.2±0.06
EC1-3 (N_DN_A)	5.8	0.41±0.005	6.1±0.05

The table shows different parameters obtained from FRET. R_0 was calculated for every donor acceptor dye pair using equation 7. Efficiency (E) values of FRET happening between donor Cy3 (N and C terminus modified) and acceptor Cy5 (N and C terminus modified) was estimated from the intensity profiles. Efficiency values decreased for N_DN_A conformation compared to N_DC_A in Cdh23 EC1-2 protein, whereas for Cdh23 EC1-3 construct both the conformations showed similar efficiency values. The distance between the dye pairs was estimated from the efficiency.

Supplementary Table 2: SAXS data collection parameters are tabulated along with the software used for analyzing the scattering data.

**Data-collection parameters**	
Instrument	SAXSpace (Anton Paar, Austria)
Beam geometry (mm^2)	Slit collimation(10mm)
Wavelength (nm)	1.5418
q range ($Å^{-1}$)	0.01 – 0.40
Temperature (K)	283
**Software employed**	
Primary data reduction	*SAXSquant*
Data processing	*PRIMUS QT*
Ab initio analysis	*DAMMIF/DAMMIN*
Validation and averaging	*DAMAVER*
Rigid-body modelling	n.a.
Computation of model intensities	*CRYSOL*
Three-dimensional graphics representations	*PyMOL*

n.a. not applicable

Supplementary Table 3: Determination of the rotational correlation decay time of Trp66 (W66) at 3 different concentrations of Cdh23 EC1-2 WT.

Rotational Correlation time	44µM Cdh23 EC1-2-2mM Ca^{2+}	11µM Cdh23 EC1-2-2mM Ca^{2+}	44µM Cdh23 EC1-2-EGTA
Φ_1	13.30±0.14 ns	11.00±0.72 ns	10.90±0.86 ns
Φ_2	609±130 ps	540±60 ps	410±63 ps

Two-exponential decay (Eq.3) showed the best fit (χ^2=0.986) to the anisotropy data (Fig 2 of main text) with the values in Table 4. Φ_1 corresponds to the global motion of W66 (molecular motion) with the protein, whereas Φ_2 corresponds to the local motion of tryptophan alone. We observed a difference in Φ_1 as we varied the concentration of the protein from monomer-dominating (11 µM) to dimer-dominating (44 µM) solutions. Φ_1 for dimers is higher, suggesting a longer tumbling time for dimers than the monomers. However, no significant change was observed for Φ_2 between 11 µM and 44 µM of proteins, suggesting a similar local environment for W66 in monomer and dimer. The rotational anisotropy decay of the proteins in Ca^{2+}-free buffer matched well with the monomeric form as expected. The little difference can be attributed due to the change in the local environment of W66 with increased flexibility in the protein in the absence of Ca^{2+}.

Supplementary Table 4: Quantitative comparison of different Patch-Dock structures

Structure	Rank	Score	Radius of gyration(Rg) nm	ΔRg (Rg Envelop - Rg Docking Structure) nm	NSD value after Supcomb	D (nm) NN	Is Difference Significant at p = 0.2
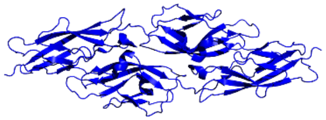	1	16284	3.20	0.30	0.71	7.7	No
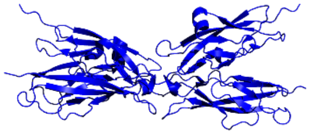	3	15664	2.90	0.60	0.79	8.7	Yes
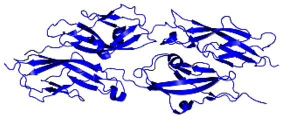	4	15612	3.00	0.50	0.74	8.7	Yes
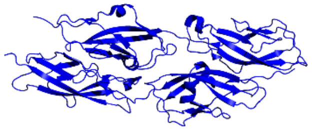	5	15444	3.07	0.43	0.76	8.8	Yes

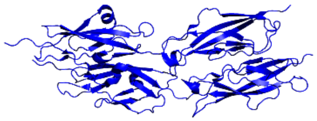	7	14918	3.02	0.48	0.74	8.7	Yes
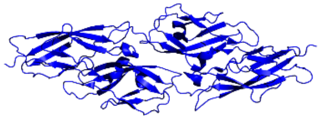	9	14342	3.10	0.40	0.76	7.8	No
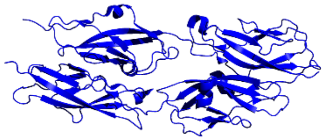	10	14328	3.04	0.46	0.77	8.7	Yes

The table shows the Rank, Score, theoretical Radius of gyration (Rg), ΔRg (R_g from SAXS – R_g from docking structure) and NSD value obtained for docking structure after superimposing with SAXS envelop using Supcomb. The ΔRg for each structure was calculated in reference to the R_g (3.52 ± 0.32 nm) calculated from SAXS experiment of Cdh23EC1+2 dimer and R_g obtained from Crysol software for each docking structure. Z-test was performed with 80% confidence interval one each R_g obtained from Crysol considering the experimental R_g value (3.52 ± 0.32 nm) obtained from SAXS

Confidence interval was calculated using:

$$Z = \frac{\bar{X} - \mu_x}{\sigma_x}$$

Where Z= 1.282 is the score at 80% (p = 0.2) confidence interval and σ_x = 0.32 is the population standard deviation.

From the above calculations, the structure with rank one was selected since the R_g calculated from Crysol correlated well with the R_g obtained from SAXS, also gave least NSD value after superimposing the structure to the SAXS envelope.

Supplementary Table 5: Parameters used during MD simulations.

System	Water Model	Force Field	Box Size (nm)	Number of Atoms	Simulation Time (ns)
Cdh23 Wild Type (STR 1, Dimer)	TIP4P	OPLS-AA	13 * 6 * 6	53000	100
Cdh23 Wild Type (without Ca^{2+}, Monomer)	TIP4P	OPLS-AA	11 * 4 * 4	23000	100

Different parameters used during MD simulations for different protein systems are tabulated. All the MD simulations were performed on GROMACS 5.0.1 using OPLS force filed. Cdh23 EC1-2 STR1 was obtained from PatchDock using PDB ID:2WHV. The same PDB was modified by removing Ca^{2+} ions for Cdh23 EC1-2 WT without Ca^{2+} ions.

Supplementary Table 6: Physical parameters of the trans-homodimer of Cdh23 EC1-2 (WT), estimated from various techniques.

Parameters	SEC	AUC	DLS	SAXS	Theoretical
**Molecular weight (Da)**	51000	52000	NA	54000	52944
**Radius of gyration, R_g (nm)**	NA	NA	NA	3.2	3.19
**Radius of hydration, R_H (nm)**	3.5*	4.1	3.9	NA	3.4

The table shows a comparison of the physical parameters, including molecular weight, the radius of gyration, and the radius of hydration of the trans-homodimer of Cdh23 EC1-2(WT). These parameters are experimentally estimated from various techniques, including SEC, AUC, DLS, and SAXS. The parameters from different experiments are comparable without any significant difference and also match with the theoretical estimates.

* **R_H** for SEC was estimated from the calibration curve of log(R_H) *vs.* V_e/V_o for standard proteins where R_H for standard proteins was estimated from independent DLS experiments.

NA: Not applicable

Supplementary Table 7: Estimation of R_g/R_H of the trans-homodimer of Cdh23 EC1-2 (WT) obtained from various methods

	**Estimated R_g/R_H ratio (R_g = 3.2 nm estimated from SAXS)**			
**Protein sample**	**SEC**	**AUC**	**DLS**	**Theoretical**
**Cdh23 EC1-2 Dimer**	0.91 (3.5)	0.78 (4.1)	0.82 (3.9)	0.94 (3.4) (SAXS envelop)

The ratio of R_g/R_H values as tabulated for the trans-homodimer of Cdh23 EC1-2 (WT) were estimated from SEC, AUC, and DLS. The R_g of 3.2 nm was estimated from the SAXS of Cdh23 EC1-2 (WT) performed at 10mg/mL (440 µM). The ratios estimated from all techniques result a value > 0.75, indicating an elongated shape of the dimer [2]. The theoretical values were measured from the SAXS envelop that perfectly fit the docked dimer structure obtained from the crystal structure (PDB ID: 2WHV). The values in the parenthesis represent R_H obtained from the respective techniques.

Supplementary Table 8: A comparison of dissociation constants and off-rate values for the first two domains of various cadherins.

**Cadherins**	$\boldsymbol{K_D}$ **(μM)** (solution)	$\boldsymbol{k_{off}}$ **($\mathbf{s^{-1}}$)** (SMFS)	**References**
E-cadherin	96.5 ± 10.6	1.09±0.35; 4.00±0.68	[3], [4]
N-cadherin	25.8 ± 1.5	0.98±0.46	[3], [4]
C-Cadherin	126.7 ± 19.7	3.9±1.54, 0.02±0.004	[5], [6]
VE-Cadherin(EC1-5)	200	1.8	[7], [8]
Dsg-1	450±41.5	5.88	[9], [10]
Dsc-2	740±24	0.23	[11], [12], [13]
Dsg-3	438±31	3.2	[14], [11]
Cadherin-23	18 ± 4	0.55;<br>$8.1*10^{-4}$	This work

References

1 Lassmann T, Frings O & Sonnhammer ELL (2009) Kalign2 : high-performance multiple alignment of protein and nucleotide sequences allowing external features. **37**, 858–865.

2 Tande BM, Wagner NJ, Mackay ME, Hawker CJ & Jeong M (2001) Viscosimetric, hydrodynamic, and conformational properties of dendrimers and dendrons. *Macromolecules* **34**, 8580–8585.

3 Katsamba P, Carroll K, Ahlsen G, Bahna F, Vendome J, Posy S, Rajebhosale M, Price S, Jessell TM, Ben-Shaul A, Shapiro L & Honig BH (2009) Linking molecular affinity and cellular specificity in cadherin-mediated adhesion. *Proc. Natl. Acad. Sci. U. S. A.* **106**, 11594–9.

4 Panorchan P (2006) Single-molecule analysis of cadherin-mediated cell-cell adhesion. *J. Cell Sci.* **119**, 66–74.

5 Vendome J, Felsovalyi K, Song H, Yang Z, Jin X, Brasch J, Harrison OJ, Ahlsen G, Bahna F, Kaczynska A, Katsamba PS, Edmond D, Hubbell WL, Shapiro L & Honig B (2014) Structural and energetic determinants of adhesive binding specificity in type I cadherins. *Proc. Natl. Acad. Sci.* **111**, E4175–E4184.

6 Bayas M V., Leung A, Evans E & Leckband D (2006) Lifetime measurements reveal kinetic differences between homophilic cadherin bonds. *Biophys. J.* **90**, 1385–1395.

7 Baumgartner W, Wendeler MW, Weth A, Koob R, Drenckhahn D & Geßner R (2008) Heterotypic trans-Interaction of LI- and E-Cadherin and Their Localization in Plasmalemmal Microdomains. *J. Mol. Biol.* **378**, 44–54.

8 Baumgartner W, Hinterdorfer P, Ness W, Raab A, Vestweber D, Schindler H & Drenckhahn D (2000) Cadherin interaction probed by atomic force microscopy. *Proc. Natl. Acad. Sci.* **97**, 4005–4010.

9 Waschke J, Menendez-Castro C, Bruggeman P, Koob R, Amagai M, Gruber HJ, Drenckhahn D & Baumgartner W (2007) Imaging and force spectroscopy on desmoglein 1 using atomic force microscopy reveal multivalent Ca2+-dependent, low-affinity trans-interaction. *J. Membr. Biol.* **216**, 83–92.

10 Harrison OJ, Brasch J, Lasso G, Katsamba PS, Ahlsen G, Honig B & Shapiro L (2016) Structural basis of adhesive binding by desmocollins and desmogleins. *Proc. Natl. Acad. Sci.* **113**, 7160–7165.

11 Harrison OJ, Brasch J, Lasso G, Katsamba PS, Ahlsen G, Honig B & Shapiro L (2016) Structural basis of adhesive binding by desmocollins and desmogleins. *Proc. Natl. Acad. Sci.* **113**, 7160–7165.

12 Lowndes M, Rakshit S, Shafraz O, Borghi N, Harmon RM, Green KJ, Sivasankar S & Nelson WJ (2014) Different roles of cadherins in the assembly and structural integrity of the desmosome complex. *J. Cell Sci.* **127**, 2339–2350.

13 Shafraz O, Rübsam M, Stahley SN, Caldara A, Kowalczyk AP, Niessen CM & Sivasankar S (2018) E-cadherin binds to desmoglein to facilitate desmosome assembly. *Elife* **7**, 1–18.

14 Spindler V, Heupel WM, Efthymiadis A, Schmidt E, Eming R, Rankl C, Hinterdorfer P, Müller T, Drenckhahn D & Waschke J (2009) Desmocollin 3-mediated binding is crucial for keratinocyte cohesion and is impaired in pemphigus. *J. Biol. Chem.* **284**, 30556–30564.
